# Contact Patterns in a High School: A Comparison between Data Collected Using Wearable Sensors, Contact Diaries and Friendship Surveys

**DOI:** 10.1371/journal.pone.0136497

**Published:** 2015-09-01

**Authors:** Rossana Mastrandrea, Julie Fournet, Alain Barrat

**Affiliations:** 1 Aix Marseille Université, Université de Toulon, CNRS, CPT, UMR 7332, 13288 Marseille, France; 2 Data Science Laboratory, ISI Foundation, Torino, Italy; National Institutes of Health, UNITED STATES

## Abstract

Given their importance in shaping social networks and determining how information or transmissible diseases propagate in a population, interactions between individuals are the subject of many data collection efforts. To this aim, different methods are commonly used, ranging from diaries and surveys to decentralised infrastructures based on wearable sensors. These methods have each advantages and limitations but are rarely compared in a given setting. Moreover, as surveys targeting friendship relations might suffer less from memory biases than contact diaries, it is interesting to explore how actual contact patterns occurring in day-to-day life compare with friendship relations and with online social links. Here we make progresses in these directions by leveraging data collected in a French high school and concerning (i) face-to-face contacts measured by two concurrent methods, namely wearable sensors and contact diaries, (ii) self-reported friendship surveys, and (iii) online social links. We compare the resulting data sets and find that most short contacts are not reported in diaries while long contacts have a large reporting probability, and that the durations of contacts tend to be overestimated in the diaries. Moreover, measured contacts corresponding to reported friendship can have durations of any length but all long contacts do correspond to a reported friendship. On the contrary, online links that are not also reported in the friendship survey correspond to short face-to-face contacts, highlighting the difference of nature between reported friendships and online links. Diaries and surveys suffer moreover from a low sampling rate, as many students did not fill them, showing that the sensor-based platform had a higher acceptability. We also show that, despite the biases of diaries and surveys, the overall structure of the contact network, as quantified by the mixing patterns between classes, is correctly captured by both networks of self-reported contacts and of friendships, and we investigate the correlations between the number of neighbors of individuals in the three networks. Overall, diaries and surveys tend to yield a correct picture of the global structural organization of the contact network, albeit with much less links, and give access to a sort of backbone of the contact network corresponding to the strongest links, i.e., the contacts of longest cumulative durations.

## Introduction

Despite the wealth of communication and interaction means made available by our modern societies, direct face-to-face interactions between individuals remain an essential element of human behavior and of human societies. They contribute to shape human social networks and determine channels of information propagation, of opinion formation, as well as the potential transmission routes of infectious diseases, in particular of respiratory pathogens. Accurate descriptions of the corresponding contact patterns represent therefore important tools in several respects: for the fundamental knowledge and understanding of human behavior and social networks, as well as to inform models of epidemic spread and to design and evaluate control measures such as the targeting of specific groups of individuals with appropriate prevention strategies or interventions.

Empirical data describing direct interactions between individuals are however by nature difficult to gather. Various techniques have been developed to this aim, and many data sets collected and exploited, in particular in the epidemiological context (see [1] for a review): surveys and diaries [2–11], synthetic population models [12–14] and, thanks to the increase in the availability and use of novel technologies, infrastructures based on various types of wearable sensors [15–27].

Methods based on surveys or diaries on the one hand, and sensing platforms based on wearable sensors on the other hand, have each advantages and limitations. Well-studied questionnaires allow to gather informations not only on the existence of contacts but also on additional characteristics, such as their context (home, work, travel), an estimate of their durations, the existence of repeated contacts with the same individual, or even the distance from home at which the contacts take place [8]. Questionnaires can also ask to specify for each contact if it involved physical contact and distinguish periods of well-being and illness of the respondent [28]. Surveys however are costly and it is difficult to recruit participants [8, 9]. Moreover, self-reporting procedures entail biases that are difficult to estimate [4, 10, 11], as participants might not recall all their contacts or might make incorrect estimates of their durations. For instance, according to [29], factors such as ambiguity, emotions, and rapid responding together with the retrospective collection of survey data can induce an *extreme responding* bias in scale-rating diaries. Furthermore, time perception can be inaccurate in retrospective analysis, as in general people’s recollections decay rapidly with time [30]; in this respect, having a precise schedule of day activity can help to assign durations to meetings [31].

Wearable sensors on the other hand can be tuned to specifically detect close-range face-to-face proximity [18, 20, 21, 27]. They afford an objective definition of contact, can detect even short encounters, and the decrease in the related costs makes nowadays large-scale deployments feasible. They also give access to temporally resolved data sets, i.e., make possible a longitudinal study of human contacts. The main limitation of automated sensing platforms based on sensors comes from the fact that they do not register contacts with individuals not participating to the data collection (not wearing any sensor) and therefore provide data on the contacts among a closed population. Sampling issues can also arise if not all the members of the population of interest agree to wear the sensors [21, 32].

Given these respective advantages and limitations of different methods, comparing data collected by both types of methods in a given population is of great interest. To our knowledge, only one such study has been performed to date, as it is rarely possible to collect data using both methods. Smieszek et al. [11] report on such a study in a high school context, showing for instance that many contacts registered by sensors are not reported in surveys, especially short ones, while long contacts are better reported. Repeating such measures and analyses in similar and different contexts is important in order to confirm such findings, which might depend on context and population, and to understand their range of validity.

Another issue of interest consists in the comparison between contact networks, corresponding to the actual behavior of individuals, and friendship relations or online social links. Many social studies are indeed performed through surveys of self-reported friendships, starting with [33], and the study of online social networks has led to the whole field of computational social science [34]. The question of how these various networks (contacts, reported friendship, online social links) overlap or complement each other is quite largely open [25, 35]. For instance, social phenomena such as homophily [36] are typically studied through questionnaires, but recent studies have shown that behavioral contact networks can also provide interesting insights in this issue [24, 37]. Combining data from different sources to obtain a more complete picture of the contacts and interactions in a population could therefore represent an interesting route to study various questions of relevance in social sciences or epidemiology.

Here we present and analyze data sets corresponding to these various types of interactions and collected through concurrent data collection methods among more than 300 high school students during one week in December 2013. High schools represent one of the numerous possible contexts in which day-to-day contacts between individuals take place, so that such data are of interest with respect to the overarching goal of gathering data sets describing contact patterns in various environments, which can for instance be used in individual-based models of realistic populations. Moreover, school and high school contexts are of particular interest in the epidemiology of infectious diseases, as young individuals typically have many contacts and can thus play an important role in the propagation of transmissible diseases. Data regarding face-to-face contacts were measured using both the SocioPatterns sensing platform [20] based on wearable sensors and contact diaries in which the students were asked to report the contacts they had. Surveys in which students were asked to nominate their friends were also used, and students were finally asked to provide the network of their contacts on Facebook. All data sets obtained using survey-like methods suffer from important sampling issues, highlighting the interest of sensor-based methods in this respect. We compare the contact patterns measured by sensors and reported by the students, in a spirit similar to the work of [10, 11]. We confirm the main findings of these previous studies, for instance that longer contacts have major reporting probabilities, and provide some additional insights: we find in particular that, despite the important discrepancies between the data obtained by both methods, the mixing patterns between classes are well identified also through contact diaries. We moreover compare the network of reported friendships and the Facebook links with the contact patterns, finding that all longest contact durations correspond to reported friendships but that reported friendship links can also correspond to short contacts. We finally perform a preliminary multiplex analysis of the various possible links between individuals: contacts, reported friendships and Facebook links. We find that reported friendships and Facebook links are not at all equivalent with respect to the durations of actual face-to-face contacts.

We make available as S1–S4 Datasets the anonymized data sets used in this paper, which can be of interest to the wide interdisciplinary research community studying complex and social networks. These files can also be downloaded from the dedicated webpage http://www.sociopatterns.org/datasets/.

## Methods

### Study design, data collection and description

The data collection concerned high school students of specific classes called “classes préparatoires” in Lycée Thiers, Marseilles, France. These classes, specific to the French schooling system, gather students for studies that take place for two years after the end of the usual high school studies. They study in a high school environment but are de facto mostly separated from the “regular” high school students: their classes are located in a different part of the high school building and they typically take their lunches separately. They constitute thus an almost closed population with few contacts with the outside world, at least during workdays. At the end of these two years, students go through competitive exams yielding admission to various higher education colleges.

The classes have different specialization: “MP” classes focus more on mathematics and physics, “PC” classes on physics and chemistry, “PSI” classes on engineering studies and “BIO” classes on biology. We collected data among students of nine classes corresponding to the second year of such studies: 3 classes of type “MP” (MP, MP*1, MP*2), two of type “PC” (PC and PC*), one of type “PSI” (PSI*) and 3 of type “BIO” (2BIO1, 2BIO2, 2BIO3). All these students must prepare a small scientific project that they present at the final exam, and several students could build a project based on their participation to the data collection, together with the use of the collected data in some small scale analysis or numerical simulations. The active involvment of some students ensured a good participation of other students to the data collection, as also reported in other works [9, 24].

We collected data of different nature.

- We deployed the contact measurement platform developed by the SocioPatterns collaboration [20], which is based on sensors that are embedded in unobtrusive wearable badges and exchange ultra-low power radio packets in order to detect close proximity of individuals wearing them. As described in detail in [20–22], the power is tuned so that the sensors can exchange packets only when within 1 − −1.5 meters of one another. Moreover, students were asked to wear the sensors on their chests using lanyards, ensuring that the devices of two individuals can only exchange radio packets when the persons are facing each other. The sensors are also tuned so that the face-to-face proximity of two individuals wearing them can be assessed over an interval of 20 seconds with a probability in excess of 99%. Contact data are thus collected with a temporal resolution of 20 seconds: two individuals are considered to be in contact during a 20*s* time window if their sensors exchanged at least one packet during that interval, and the contact event is considered over when the sensors do not exchange packets over a 20*s* interval. The information on face-to-face proximity events detected by the wearable sensors is relayed to radio receivers installed throughout the high school: contacts occurring outside the school premises were not measured. Thanks to this infrastructure, we gathered contact data of the 327 participating students (out of 379 in the 9 classes, i.e., a 86.3% participation rate) during the week of Dec. 2-6, 2013.
- At the end of one specific day, namely Dec. 5, 2013, we asked students to fill in paper contact diaries: they were asked to give the list of other students they had had contact with (where contact was defined as close face-to-face proximity) during the day in the high school, and to give the approximate aggregated duration of the contacts with each nominated individual, to choose in one of four possible categories: at most 5 minutes, between 5 and 15 minutes, between 15 minutes and 1 hour, more than one hour. 120 students returned a filled in diary.
- During the period of the deployment, we moreover asked the students to fill in a survey in which they were asked to give the names of their friends in the high school. We obtained such friendship surveys from 135 students.
- We asked students to use the Netvizz application (https://apps.facebook.com/netvizz/) to create their local network of Facebook friendships (i.e., the use of the application by a student yields the network of Facebook friendship relations between this student’s Facebook friends). 17 students gave us access to their local network, from which we removed all users who were not concerned by the data collection.
- Each participating student gave us moreover access to the following metadata: gender, class, class in the previous year, whether s/he was smoker, and if s/he was repeating the year.

We finally note that the present data collection was the third of this kind in this high school. The data coming from the previous two data collections (performed in Dec. 2011 and Nov. 2012) were analyzed in [24]. They concerned only the contacts recorded by the SocioPatterns infrastructure (for three classes in 2011 and five classes in 2012), but no contact diaries nor friendship surveys.

### Ethics and privacy

Before the study, students and teachers were informed on the details and aims of the study. A signed informed consent was obtained for each participant (no minors were involved as all students were at least 18 at the time of the deployment). They received a wearable sensor to wear during the school time. No personal information, besides the ones indicated in the previous section, were collected. The *Commission Nationale de l’Informatique et des Libertés* (CNIL, http://www.cnil.fr), representing the French national bodies responsible for ethics and privacy, approved the study as well as the high school authorities (Proviseur du Lycée Thiers).

### Data analysis

The collected data sets have different nature and different resolutions. The most detailed data comes from the SocioPatterns infrastructure and consists in a temporal network of contacts between students, with a temporal resolution of 20 seconds: the nodes of this network represent students, and for each time window of 20 seconds a link is drawn between pairs of students between whom contacts are detected. This temporal network can be aggregated temporally over a given duration, for instance a day or the whole duration of the data collection: weighted daily or global aggregated networks are then obtained. In each aggregated network, a node represents an individual and a weighted link between two nodes *i* and *j* represents the fact that the two corresponding individuals have been in contact at least once during the aggregation time window. The weight *w*_*ij*_ of a link between *i* and *j* is given by the total time spent in contact by *i* and *j* during the aggregation window. The data can be further aggregated by grouping together students of the same class: the resulting mixing patterns between classes are described by the so-called “contact matrices”. We define contact matrices of different types:

- in the contact matrix of link densities, the element *X*, *Y* is given by the density of links between classes *X* and *Y*, i.e., the number of links *E*_*XY*_ between individuals of class *X* and individuals of class *Y* normalized by the maximum possible number of such links (*n*_*X*_ *n*_*Y*_ if *X* ≠ *Y*, *n*_*X*_(*n*_*X*_ − 1)/2 if *X* = *Y*, where *n*_*X*_ is the number of students in class *X*).
- in the contact matrix of contact durations, the element *X*, *Y* is defined as *W*_*XY*_ = ∑_*i*∈*X*,*j*∈*Y*_ *w*_*ij*_ (and *W*_*XX*_ = ∑_*i*,*j*∈*X*_ *w*_*ij*_/2): it gives the total time spent in contact between students of class *X* and students of class *Y*. These elements can also be normalized, e.g., *W*_*XY*_/*n*_*X*_ gives the average time spent by a student of class *X* with students of class *Y*.

As for the aggregated networks, these matrices can be defined on any temporal aggregation time-window.

The contact diaries, on the other hand, do not provide temporally resolved data. As each participating student reports contacts with other students, giving a certain aggregated duration for each, the resulting data set is a weighted directed network: in this network, each node is a student and a link is drawn from *i* to *j* with weight $w_{ij}^{diary}$ if student *i* reported contacts of total duration $w_{ij}^{diary}$ with student *j*. Note that links are not necessarily reciprocated, i.e., student *j* might not report a contact with *i* even if *i* reported such a contact and, even when they are reciprocated, the reported durations might not coincide, i.e., $w_{ij}^{diary}$ is not necessarily equal to $w_{ji}^{diary}$. We will first perform a systematic study of these discrepancies. Then, in order to compare the contact diary data with the sensor data as in [11], we will consider a symmetrized version of the network, in which a link exists between *i* and *j* if at least one of the two students reported a contact, and the weight of the link is taken as the maximum of $w_{ij}^{diary}$ and $w_{ji}^{diary}$. From such symmetrized network, we can also aggregate the data by class and obtain contact matrices.

The friendship survey yields, as the contact diaries, a directed network between students: indeed, a student *i* might nominate *j* as a friend without being nominated by *j*. As we did not ask students to quantify the intensity of their friendships, we obtain a directed unweighted network of reported friendship relations. We can also symmetrize the network in order to compare it with the aggregated contact network, and obtain a link density contact matrix between classes.

**Fig 1:**
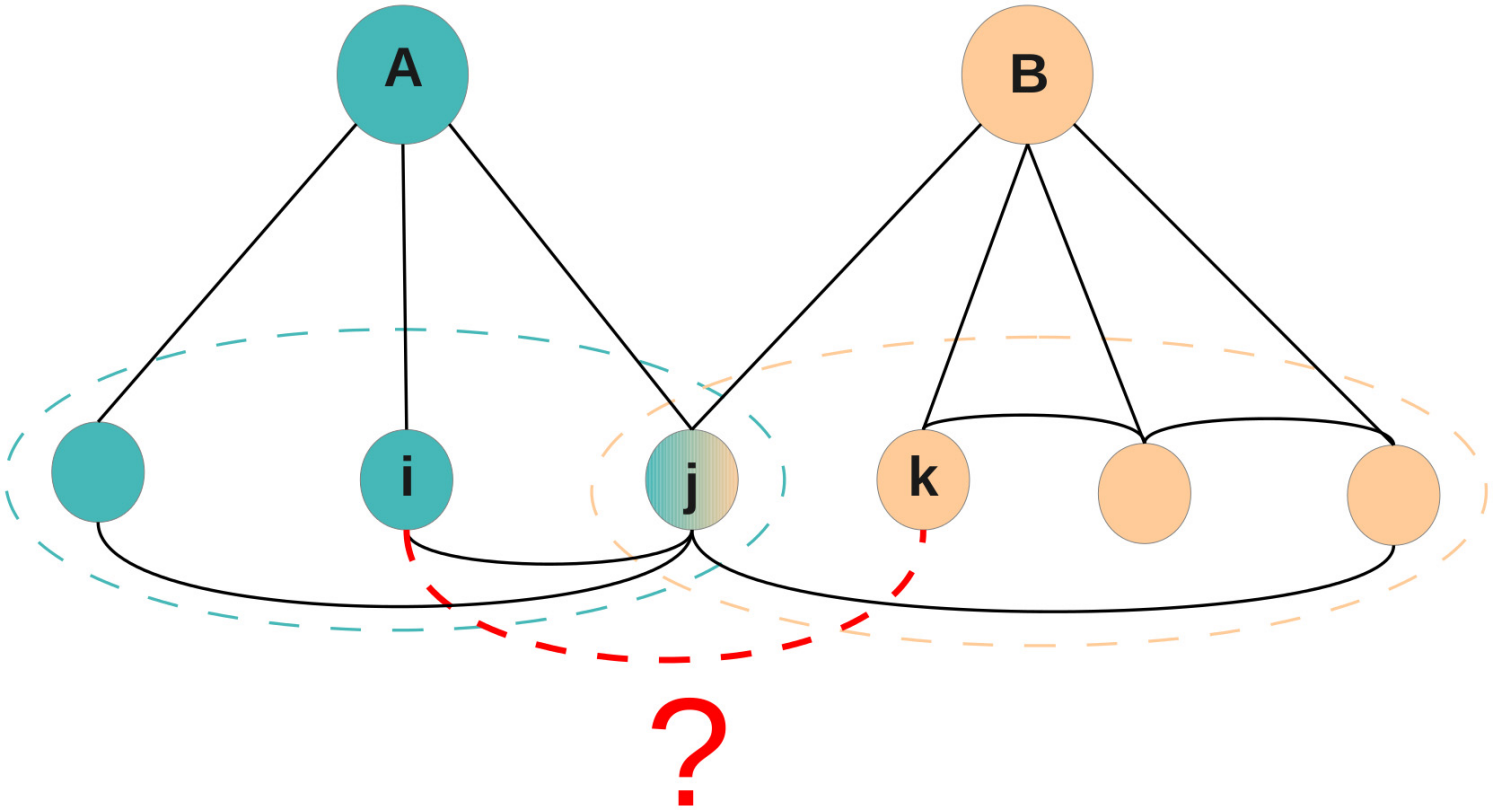
Facebook local networks. The local Facebook friendship networks provided by students *A* and *B* are shown in black. In particular, we know that *i* and *j* are friends on Facebook but not *j* and *k*, as *i* and *j* are both friends of *A* and *j* and *k* are both friends of *B*. On the other hand, we do not know if *i* and *k* are friends or not: the red dashed line represents the lack of knowledge about the potential existence of this relationship.

Finally, the data set gathered from the local Facebook friendship networks has a slightly more complex character. Indeed, for each individual who gave us access to his/her local network, we obtain the Facebook friendship links between his/her Facebook friends. However, as only few students provided us with such data, the presence or absence of a Facebook friendship link between many pairs of students remained unknown. To understand this point, let us take the example of two students *A* and *B*, with sets of Facebook friends *F*_*A*_ and *F*_*B*_. Let us now take two students *i* and *j* in the union *F*_*A*_ ∪ *F*_*B*_. If both *i* and *j* are in *F*_*A*_, or both are in *F*_*B*_, we know if they are Facebook friends or not. If however *i* ∈ *F*_*A*_∖*F*_*B*_ and *j* ∈ *F*_*B*_∖*F*_*A*_, i.e., *i* is friend with *A* but not with *B* and *j* is friend with *B* but not with *A*, we do not have access to the existence or absence of a friendship link between *i* and *j* (see Fig 1). As a result, the data set can not be represented as a network but consists in a list of pairs of students for which we know if they are Facebook friends or not (list of “known-pairs”) and a list of pairs for which the presence or absence of such a link is unknown.

## Results

**Fig 2:**
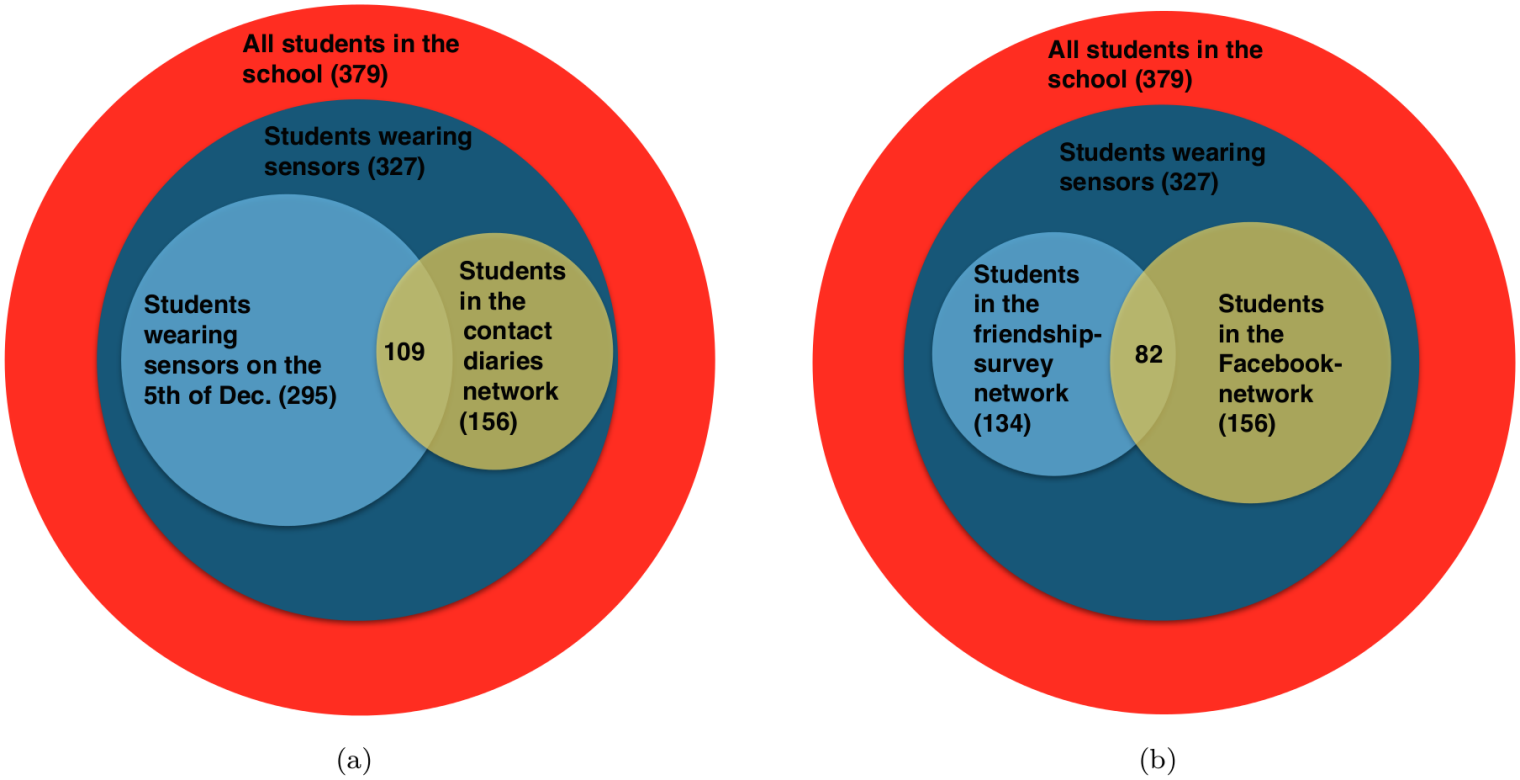
Venn diagrams representing the sets of students concerned by the various data collection efforts.

Not all students participated to the data collection effort: out of the 379 students in the classes of interest, 327 wore sensors, 120 filled in a contact diary and 135 answered the friendship survey. Moreover, the Facebook data we collected concerned 156 students. We show in Fig 2 the corresponding Venn diagrams.

### Contact patterns from sensor data

During the data collection, 67,613 contact events were registered between the 327 students carrying wearable sensors, with a cumulative duration of 3,770,160s (∼ 1047 hours). As the statistical characteristics of these contacts are very similar to the ones obtained in the previous data collections in the same setting [24], we only provide here a summary of these results and refer the interested reader to S1 Supporting Information for details.

As commonly observed in such data [38, 39], the duration of contacts was highly variable: the average duration of a contact was 56 seconds, with however 75% of the contacts having duration smaller than 1 minute and on the other hand 2% lasting more than 5 minutes. The distribution of these durations, shown in S1 Supporting Information, is very broad, spanning several orders of magnitude, with a coefficient of variation (CV) equal to 2.7. Moreover, the time intervals between successive contacts are also broadly distributed, spanning several orders of magnitude: most intercontact durations are short, but very long durations are also observed, and no characteristic timescale emerges [15, 18, 21, 39–41].

The contact network aggregated over the whole data collection has 327 nodes and 5818 weighted edges. As expected in such networks, the average shortest path length (SPL) is small (2.16) and the clustering coefficient is large (≈ 0.5, against ≈ 0.11 in a random network with the same number of nodes and edges). As also found in other similar data sets [22, 24], the distribution of degrees (the degree of a node is the number of other nodes it is connected to) is narrow (*CV* ≈ 0.38). The average degree, i.e., the average number of students each student had contact with, is ≈ 35. On the other hand, the distribution of links weights, i.e., of the total time spent in contact by pairs of students, is broad: the average cumulated duration is of 648 seconds (∼ 11 minutes), but most weights are much smaller (59% of the links have a weight smaller than 2 minutes) and large values are also observed (12% of the weights are larger than 15 minutes and 4% are larger than 1 hour). Overall, the distribution spans several orders of magnitude (CV = 3.87) and no characteristic interaction timescale can be naturally defined.

The population of interest is structured into 9 classes. We compute the contact matrices defined above to describe the mixing patterns between these classes (see S1 Supporting Information). As also observed in [24], most contacts occur within classes, and students of different classes have very few contacts: 62,342 contacts (92.2% of the total), representing a cumulated duration of 3,505,380*s* (93% of the total contact time of students), were recorded between pairs of students belonging to the same class. An additional substructure of three groups of three classes each emerges moreover: (i) classes MP, MP*1, MP*2, (ii) classes PC, PC*, PSI*, (iii) classes 2BIO1, 2BIO2, 2BIO3. More contacts are observed between students of two classes in the same group than between students of two different groups.

Nine of the students were actively involved in the data collection as they then used the data for scientific projects. These students belonged to different classes: 3 to 2BIO3, 2 to MP, 2 to MP*2, 1 to PC, 1 to PSI*. We have verified that their contact characteristics are spread over the whole range of observed values for the other students, in terms of numbers or duration of contacts as well as in terms of centrality in the aggregated contact network.

We finally mention that the number of contacts fluctuates strongly throughout the day, along a robust daily pattern driven by the occurrence of class breaks and lunches. As in [24], the daily contact matrices are very similar to each other (with cosine similarities between matrices ranging from 92.7% to 98%). Overall, although the contacts occurring in different days are not all the same, the temporal daily fluctuations and the mixing patterns between classes are robust across different days.

### Contact diaries

As mentioned above, the contact diaries yield a directed weighted network of reported contacts between students. Only 120 students filled in a contact diary, yielding a network of reported contacts of 120 nodes and 502 directed, weighted edges among them. Note that we ignore the contacts reported with students who did not fill a diary. Moreover, in the following we consider only the 295 students for whom the sensors also registered contact data during the day concerned by the contact diary. We finally obtain 109 students for whom we have both sensor and diary data, and the resulting contact diary network has 416 directed weighted links.

158 contacts were reported by only one student (non-reciprocated links), while the reciprocated links correspond to 129 pairs of students reporting both a contact with each other. Moreover, reciprocated links were sometimes reported with different durations by the two students involved. Table 1 gives the corresponding statistics. In 81 cases out of 129, both students involved reported the same duration category. In 35 cases, the reports of the two involved students differed by only one category. These results are similar to the ones of [10]. Following [10], we moreover compute the probability *P* to report a contact of a certain duration, under the hypothesis that such probability depends only on the duration. If *N*_*c*_ is the real number of contacts, and *N*_*both*_ is the number of pairs of students reporting both the contact, then *N*_*both*_ = *N*_*c*_ *P* ^2^, while the number of contacts reported by only one student is *N*_*one*_ = 2*N*_*c*_ *P*(1 − *P*); as a result, the estimate of *P* is given by *N*_*both*_/(*N*_*both*_ + *N*_*one*_/2). We obtain that the overall reporting probability is *P* ≈ 62%. Assuming that the correct duration of a reported contact is the highest reported value, we obtain that the probability to report a contact is 40% for contacts of less than 5 min, 54% for contacts between 6 and 15 min, 61% for contacts between 15 and 60 min, and 72% for contacts with aggregate duration longer than one hour.

**Table 1:** Cross-tabulation of pairs of contact reports from the contact diaries. Each pair of participants with at least one contact reported gives a single observation. For instance, there were 12 pairs of students (*i*, *j*) such that *i* reported contacts with *j* with total duration between 6 and 15 min while *j* reported a duration between 15 min and 1 h. Each percentage within a cell represents the percentage with respect to the row (right of the cell entry) and column (below the cell entry) totals.

Reported duration: higher value					
Reported duration: lower value	less than 5 min	5-15 min	15-60 min	more than 60 min	Row Tot
**Not reported**	**38** (24%)	**31** (20%)	**33** (21%)	**56** (35%)	**158** (100%)
	(75%)	(63%)	(56%)	(44%)	(55%)
**less than 5 min**	**13** (42%)	**10** (32%)	**5** (16%)	**3** (10%)	**31** (100%)
	(25%)	(21%)	(9%)	(2%)	(11%)
**5-15 min**		**8** (32%)	**12** (48%)	**5** (20%)	**25** (100%)
		(16%)	(20%)	(4%)	(9%)
**15-60 min**			**9** (41%)	**13** (59%)	**22** (100%)
			(15%)	(10%)	(8%)
**more than 60 min**				**51** (100%)	**51** (100%)
				(40%)	(17%)
**Column Tot**	**51** (18%)	**49** (17%)	**59** (20.5%)	**128** (44.5%)	**287** (100%)
	(100%)	(100%)	(100%)	(100%)	(100%)

### Comparing contact diaries and sensor data

In this section, we compare the data collected by the wearable sensors with the contacts reported by the students using contact diaries. We therefore consider on the one hand the weighted network of the contacts registered by the sensors on Dec. 5^*th*^, and on the other hand the symmetrized version of the network obtained from the contact diaries, in which the highest value of the aggregated contact duration reported by two students is retained.

Given many students did not fill in the diaries, we first compare the sensor contact data of respondents and non-respondents. Respondents were not uniformly distributed in classes: 20 were in 2BIO1, 11 in 2BIO2, 13 in 2BIO3, 23 in MP, 1 in MP*1, 18 in MP*2, 23 in PC, none in PC* nor PSI*. This has consequences on the overall structure of the network (see below) as some classes are not or almost not represented. On the other hand, we have compared the properties (numbers, durations and aggregate durations of contacts, degree and centrality in the aggregated contact network) of respondents and non-respondents using Wilcoxon tests and did not find any significant difference.

Table 2 reports some properties of these networks. As not all students filled in contact diaries, we moreover report the networks’ properties when restricted to the nodes present in both (109 nodes). The density and average degree of the contact network obtained by the sensors are almost twice as large as the ones obtained using contact diaries, but the degree distributions have similar shapes (see S1 Supporting Information). The cliques are also larger in the sensor contact network, while the average shortest path length is smaller (the distributions of shortest path lengths is shown in S1 Supporting Information): nodes seem farther apart in the contact diary network than in the sensor data network. The average clustering on the other hand is similar.

**Table 2:** Comparison of properties for the contact networks obtained from sensors and diaries. On the day of collection of the contact diaries, only 295 students out of the 327 participating correctly wore their sensors. All network properties for the contact diaries network are computed on its symmetrized version. In this summary table we assume that if a contact is reported by at least one of the two nodes, it exists. The right side of the table is performed after matching the two networks. Matching is done by removing the nodes who did not participate to the survey and the ones who did not have contacts recorded by sensors on the 4th day of the study. A * close to the SPL average means that after the match some isolated nodes appeared. In this case, we computed the average on the connected pairs only. Standard deviations are given in parentheses.

	Sensors	Contact diaries	Sensors	Contact diaries
**Nodes**	295	120	109	109
**Links**	2162	348	488	287
**Density**	0.05	0.05	0.08	0.05
**Avg. Degree**	15(8)	6(2)	9(4)	5(2)
**Avg. Clustering**	0.38(0.18)	0.45(0.25)	0.45(0.21)	0.44(0.26)
**Avg. SPL**	2.81(0.8)	5.36(2.73)	2.94*(1.03)	5.36(2.66)
**Maximal Clique**	9	5	8	5

**Fig 3:**
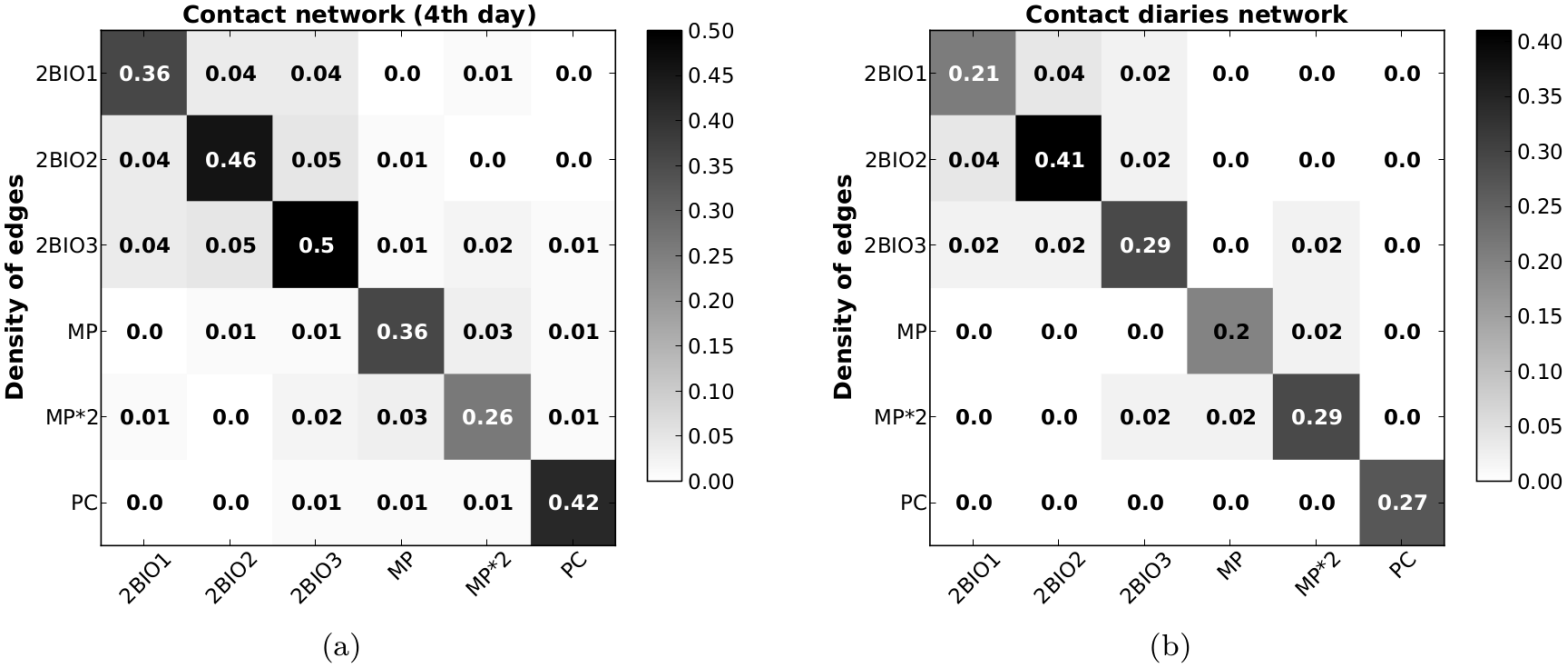
Contact matrices of link densities. We compare here the contact matrices of link densities built from (a) the network of contacts obtained using the sensor data collected on Dec. 5^*th*^ and (b) the network of contacts as reported in the contact diaries. We discarded here the data corresponding to the MP*1, PC* and PSI* classes as too few students from these classes filled in a contact diary (2 for MP*1, 0 for PC* and PSI*). The similarity between these two matrices is of 97%.

Interestingly, the strongly structured character of the contact network, as highlighted by the dominance of the diagonal elements of the contact matrices and the existence of groups of classes, is well preserved in the contact diary network, as shown in Fig 3. A high similarity is obtained between the link density contact matrices computed on both networks: despite the low sampling of the contact diaries, a sensible information on the mixing patterns between classes is obtained.

It is clear from the different numbers of links in the two networks that there are discrepancies between sensor-based data and contact diaries, as also found in [11]. Overall, 70.4% of the links obtained from contact diaries correspond to contacts registered by the sensors, while only 41.4% of the contacts registered by the sensors find a match in the contact diary. We now investigate these discrepancies in more details.

**Fig 4:**
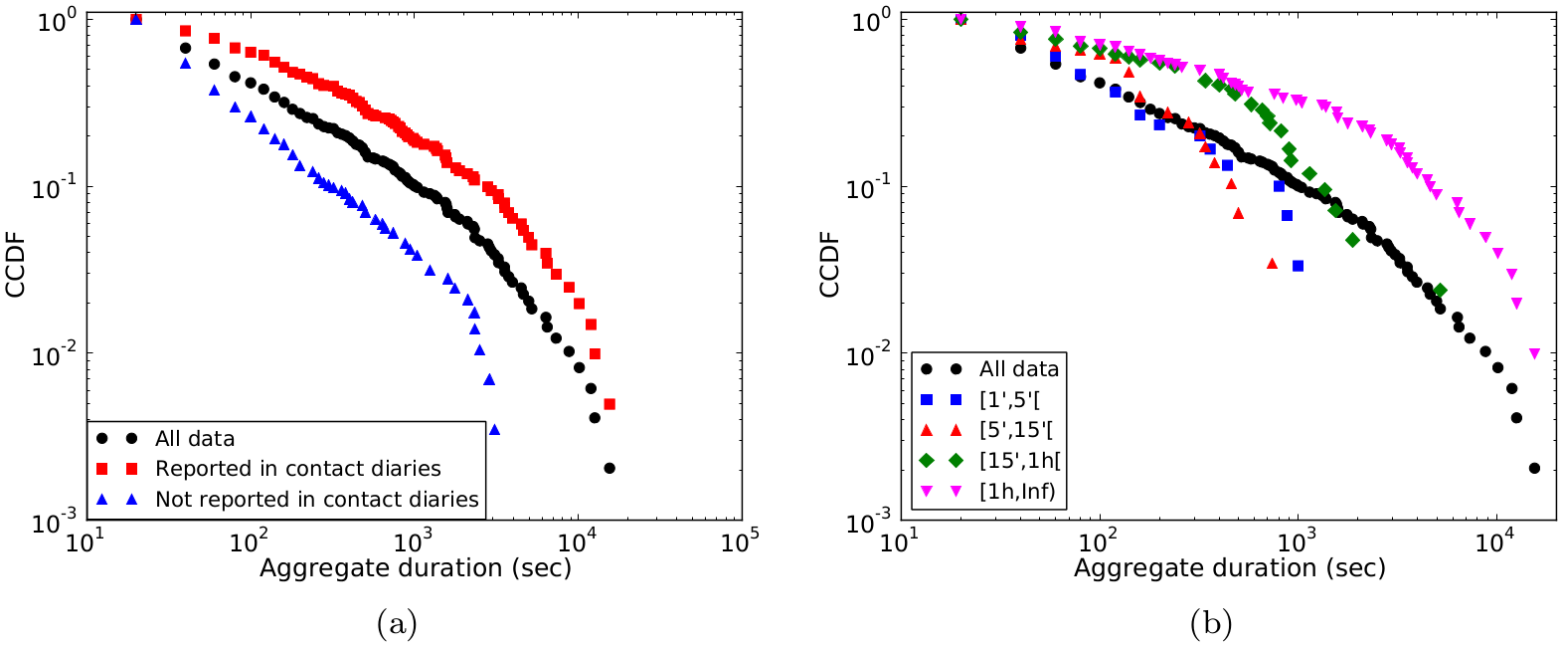
Sensors vs. contact diaries: distributions of cumulative durations registered by the sensors. (a) Cumulative distributions of the aggregate durations of contacts registered by the sensors for (i) all 488 links between the 109 nodes belonging to both networks; (ii) the 202 links that were also reported in the diaries; (iii) the 286 links that were not reported in the diaries. (b) Cumulative distribution of aggregate durations of contacts registered by the sensors for the different categories of links reported in the diaries.

Fig 4(a) and Table 3 first compare the distributions of the cumulative durations of the links registered by the sensors, distinguishing between the links which were reported in the contact diaries and those which were not. For reference, the figure also reports the distribution of durations for all the links registered by the sensors. Both distributions are broad, spanning several orders of magnitude. However, the distribution of durations for the links finding a match in the contact diaries is much broader, with much larger average duration and standard deviation (Wilcoxon tests for each pair of distributions reject the null hypothesis of equality of the distributions). In particular, links not reported tend to correspond to smaller durations, and all the links with a duration above a certain threshold (close to 1 hour) were reported in the diaries.

**Table 3:** Average and standard deviation of the distributions of aggregate durations for different sets of links (as in Fig 4).

	All data	Links present in the contact diaries	Links absent from the contact diaries
**Mean (s)**	480	934	156
**Std. dev.**	1470	2152	413
**#links**	488	202	286

We moreover investigate in Fig 4(b) the diversity of the cumulative durations registered by the sensors for the links reported in the diaries in each category. Strikingly, all distributions are rather broad and, given a reported category, both much shorter and much longer durations can be registered by the sensors. In particular, the distributions corresponding to the two first categories (less than 5 min and between 5 and 15 min) are similar. However, the distributions become consistently broader for categories corresponding to larger durations, and durations (as registered by the sensors) above a certain threshold are reported only in the highest duration category of the diaries.

Table 4 gives more details through a cross-tabulation of the aggregate durations of the contacts as registered by the sensors or reported in the diaries. If we consider the duration registered by the sensors as accurate, we obtain that 68% of the contacts in the first category (1–5 min) were not reported, against 29% and 31% for the next categories, while all the contacts lasting more than 1 hour were reported.

**Table 4:** Sensors vs. contact diaries: cross-tabulation of the number of links in each duration category. The percentages within a cell are computed with respect to the row (right of the cell entry) and column (below the cell entry) totals.

SENSORS						
SURVEY	Not detected	< 5 min	5-15 min	15-60 min	> 60 min	Row Tot
**Not reported**	**unknown** (n/a)	**258** (90%)	**16** (5.5%)	**12** (4.5%)	**0** (0%)	**286** (100%)
	(n/a)	(68%)	(29%)	(31%)	(0%)	(53%)
****<** 5 min**	**36** (44%)	**38** (46.5%)	**7** (8.5%)	**1** (1%)	**0** (0%)	**82** (100%)
	(42%)	(10%)	(12.5%)	(3%)	(0%)	(11%)
**5-15 min**	**5** (28%)	**9** (50%)	**4** (22%)	**0** (0%)	**0**(0%)	**18** (100%)
	(6%)	(2%)	(7%)	(0%)	(0%)	(5%)
**15-60 min**	**17** (29%)	**24** (40.5%)	**12** (20%)	**5** (8.5%)	**1** (2%)	**59** (100%)
	(20%)	(6%)	(21.5%)	(13%)	(7%)	(10%)
****>** 60 min**	**27** (21%)	**51** (40%)	**17** (13%)	**20** (16%)	**13** (10%)	**128** (100%)
	(32%)	(13.5%)	(30%)	(53%)	(93%)	(22%)
**Column Tot**	**85** (15%)	**380** (66%)	**56** (10%)	**38** (7%)	**14** (2%)	**573** (100%)
	(100%)	(100%)	(100%)	(100%)	(100%)	(100%)

Among the 202 links found in both networks, discrepancies emerge moreover between the durations reported by the students and registered by the wearable sensors. In particular, 60 (29.7%) links correspond to the same duration category in both cases, while 133 (65.8%) are overestimated in the diaries with respect to the sensor data, and only 9 (4.5%) are assigned a shorter duration in the diaries than in the sensor data (overall, the Kendall’s *τ* computed for the list of links ranked according to the durations either registered or reported yields a rank-correlation of ≈ 45%). Note that, if we use a symmetrized version of the contact diary network in which we retain the lowest value of the aggregated duration of contacts reported by each pair of individuals (including 0 for links non-reported by one of the individuals) instead of the highest, the number of links common with the network obtained from sensor data drops to 102, but the other results are robust. In particular, 32 links (31%) correspond to the same duration category in both networks, 64 (63%) to an overestimation in the diaries, and 6 (6%) to an underestimation.

We finally investigate the discrepancies between sensor data and diaries at the individual level. In contrast with the data reported in [11], we observe a significant correlation (0.4) between the degree of the nodes in the two networks, despite important fluctuations are present (see S1 Supporting Information). More in detail, and when considering in addition the directed character of the network built from the diaries, we do not find any significant correlation between the out-degree of a node *i* in the diaries’ network (number of contacts reported by the corresponding student) and its degree in the sensor data network ($k_{i}^{sensors}$). However, we find a significant positive correlation (equal to 0.4) between $k_{i}^{sensors}$ and the in-degree of *i* in the diaries’ network, which represents the number of other students who have reported a contact with him/her. Similar correlation values are obtained when considering only contacts larger than a given threshold (see S1 Supporting Information).

We refine this result by investigating in more detail the relationship between out-degree in the contact diary network and the degree in the sensor data network. We start from the idea, supported by the evidence provided above, that among contacts of different durations individuals tend to record more easily their longest contacts in a contact diary. We then compute for each student the coefficient of variation (*CV*) of the longest durations of his/her encounters with other students, which gives the extent of the variability of these durations: *CV*_*i*_ ≤ 1 means that the student *i* has contacts of similar durations with other students, while *CV*_*i*_ > 1 corresponds to a large variability, i.e., that *i* divides his/her contact time in a heterogeneous way among the other students s/he has met during the day. No particular grouping of individuals with *CV*_*i*_ > 1 was observed with respect to the various features (gender, class, field of study) of the students. We obtain 42 students with *CV* ≤ 1 and 67 with *CV* > 1, and a significant correlation (close to 0.35) between the out-degree in the contact diary network and $k_{i}^{sensors}$ in the first group, but no significant correlation in the second group (the correlation for the in-degree is present and around 0.4 in both groups). In other words, for students who have encounters of similar maximum durations with other individuals (*CV* ≤ 1), the contact diary data reported by these students correlate with the data registered by the sensors. For students whose maximum contact durations are heterogeneous (*CV* > 1) on the other hand, no correlation in the diary-reported and sensor-measured degrees is observed. This is perfectly in line with our initial hypothesis: For *CV* ≤ 1 the number of links remembered is then correlated with the real number of links in the contact network while, if *CV* > 1, there might be an arbitrary large number of links with only short contacts that are not remembered in the diaries: in the latter case, the number of links that are reported is not correlated with the total number of links registered by the sensors.

### Multiplex network of students’ relationships

The face-to-face proximity measured by different methods as described in the previous sections represent only one type of relationship between individuals. Other social ties exist, such as friendships and online relationships, and contribute to form a multiplex network in which the nodes represent students and links of different nature coexist between them. These links might a priori be related (one has more contacts with a friend, or becomes friend with someone after meeting him/her often, etc…) but might also differ substantially: one can be very good friend with someone and meet him/her only rarely because of specific constraints (such as different schedules of classes). It is thus of interest to compare the different layers of this multiplex network and to investigate what information on actual contacts can be gathered from data describing friendship relations. In particular, friendship survey data might be more reliable than contact diaries: it might be easier to remember the names of one’s friends than the contacts occurred during a day and moreover, friendships evolve on slower timescales so that friendship surveys can more easily be gathered on several days without memory biases.

**Fig 5:**
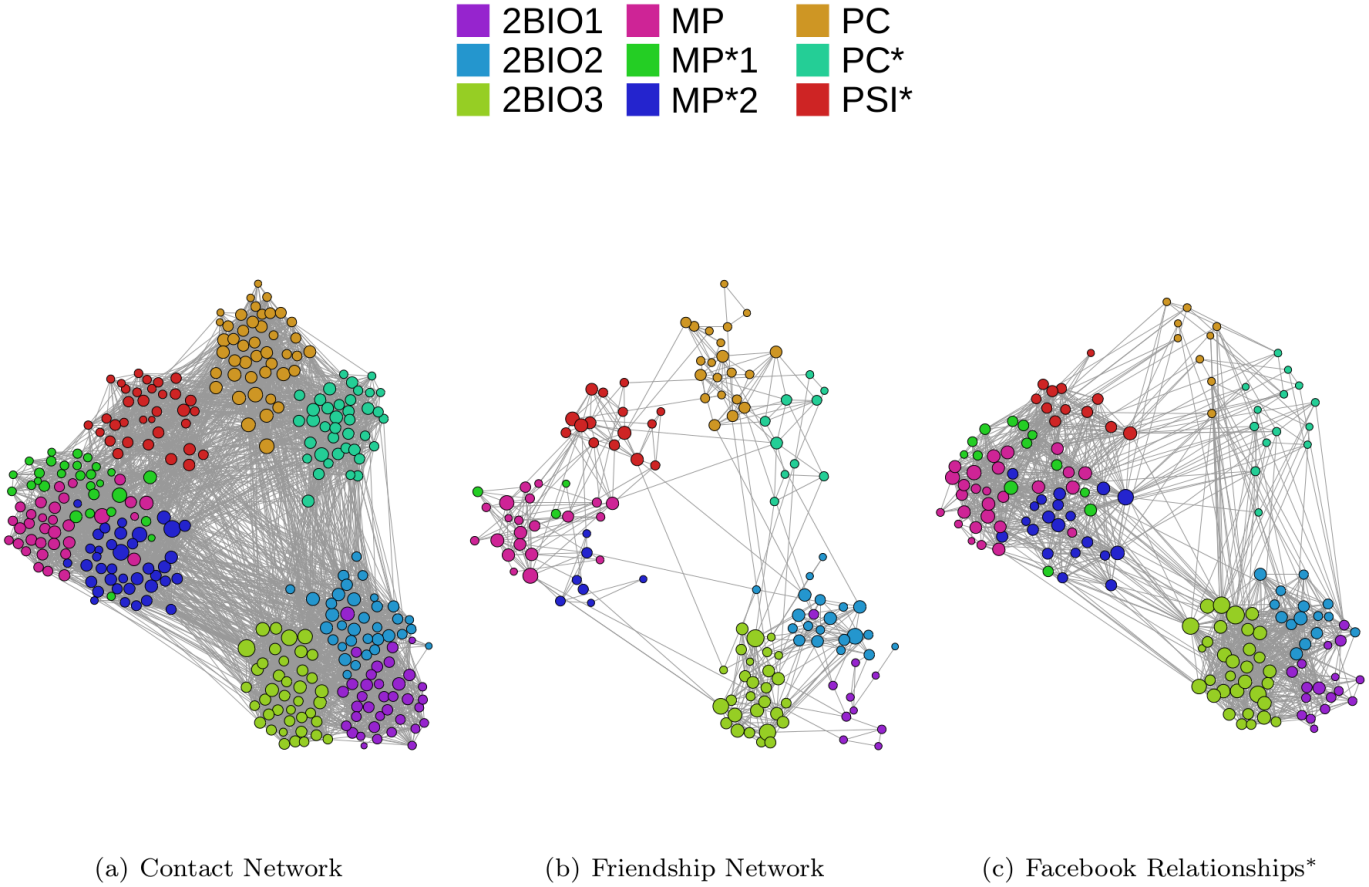
Contact and friendship networks. The three layers of the multiplex are shown using exactly the same layout: each node is placed at the same position in the three panels. The color of each node represents its class and size represents its degree in the corresponding network (here we consider a symmetrized version of the network of reported friendships). * Strictly speaking, the Facebook data do not provide a network as we do not have information about the presence or absence of a link between many pairs of nodes (see Fig 1). Figure created using the Gephi software http://www.gephi.org.

As described in the Methods section, we have collected data concerning the friendships and online Facebook links of a number of students, and we compare in this section the properties of the resulting data sets. Fig 5 displays the network of contacts registered by the sensors during the week of data collection, as well as the network of reported friendships and the Facebook links, using the same position for the nodes in the three representations (as explained in the Methods section, in the case of Facebook other links than the ones represented might exist: we are only representing the “known-pairs”, so that Fig 5 might be an underestimation of the real number of existing Facebook links. For this reason, standard network metrics cannot be computed in this case). The friendship survey and Facebook data involve respectively 41% and 48% of the students who participated to the data collection, so that the resulting networks have also clearly much less links than the contact network. Overall, the networks appear substantially different, although the grouping of nodes in classes seems relevant in all three cases. We perform a more detailed comparison in the next paragraphs.

#### Respondents vs. non-respondents

The students who filled in the friendship survey were not spread evenly in the various classes: 11 were in 2BIO1, 18 in 2BIO2, 28 in 2BIO3, 21 in PC, 3 in MP*1, 7 in MP*2, 21 in PC, 10 in PC*, 15 in PSI*. However, as in the case of the contact diaries, we have checked using Wilcoxon tests that respondents and non-respondents do not show significant differences in their contact characteristics (durations, aggregate durations, degree and centrality in the contact network measured by the sensors).

#### Contact network versus friendship-survey network

**Fig 6:**
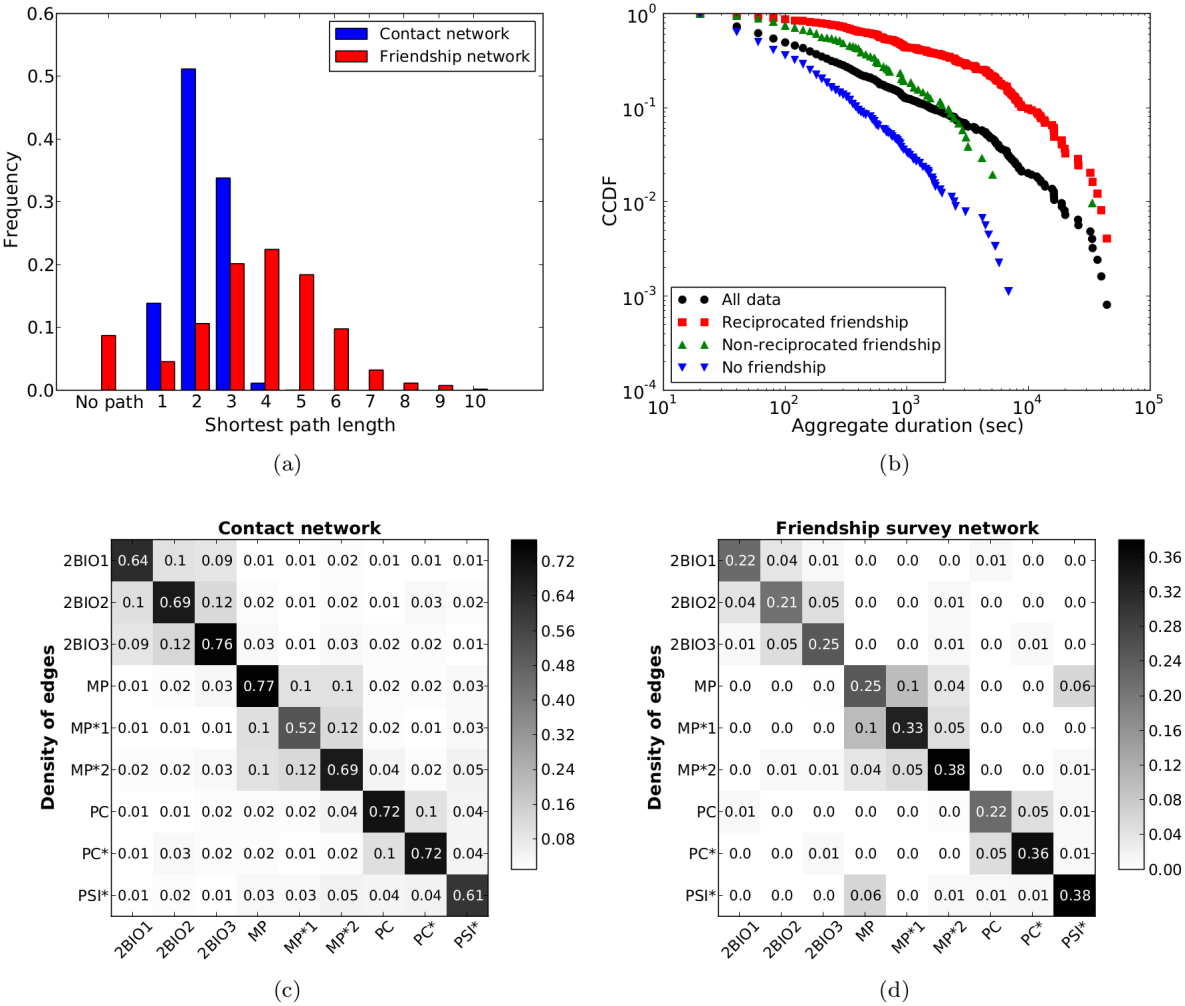
Comparison of the networks of contacts and friendships. (a) Shortest path length distributions for both networks; (b) Distributions of aggregate durations, as measured by the sensors, for different kinds of links in the contact network: (i) all links, (ii) links *i* − *j* for which only one of *i* or *j* reported a friendship with the other, (iii) links for which both students reported the friendship, and (iv) links for which no friendship was reported; (c) and (d): Contact matrices of link densities. We compare here the contact matrices of link densities built from (c) the global aggregated network of contacts obtained using the sensor data and (d) the symmetrized network of reported friendships. The similarity between these two matrices is ≈ 95%.

As for the contact diaries, the network built from the friendship surveys is directed: a student *A* might report another student *B* as a friend while *B* does not mention *A*. In the present data set, the network comprises 689 directed links of which 137 are not reciprocated (and 276 pairs of students declare a friendship towards each other). In the following, we will for the sake of simplicity mostly consider a symmetrized version of the network in which a link is drawn between two students if at least one of them has reported a friendship with the other. The resulting network has 135 nodes and 413 links. Table 5 reports a comparison between the main features of the networks of reported friendships and of contacts. Here we consider the network of contacts registered by the sensors, aggregated over the whole data collection, and we report the properties of the whole networks and of the networks once restricted to the same set of nodes (as many students who wore a sensor did not fill in the friendship survey). Similarly to what we found in the contact diary data, we observe a much denser network for the sensor data than for the friendship network (with narrow degree distributions, see S1 Supporting Information). This is quite expected as one naturally encounters many persons whom one would not list as friends in a survey. The contact network has as well larger cliques and smaller shortest path lengths, as shown in Fig 6(a): most pairs of students are only at distance 2 in the contact network, and the maximal distance is 4, while pairs of students can be separated by as much as 10 hops in the network of friendships.

**Table 5:** Summary statistics for the global contact network and the network of reported friendships. All network properties for the network of friendships are computed on its symmetrized version. Values on the right part of the table are obtained after retaining only the students present in both networks.

	Contact network	Reported Friendships	Contact network	Reported Friendships
**Nodes**	327	135	134	134
**Links**	5818	413	1235	406
**Density**	0.11	0.05	0.14	0.05
**Avg. Degree**	36(13)	6(3)	18(8)	6(3)
**Avg. Clustering**	0.5(0.14)	0.53(0.29)	0.55(0.18)	0.54(0.29)
**Avg. SPL**	2.16(0.6)	4.06*(1.6)	2.22(0.69)	4.02*(1.6)
**Largest clique size**	23	8	14	8

* The network of friendships is not connected so the average is computed taking into account only the pairs of nodes belonging to the same connected component. Numbers in parentheses represent the standard deviations.

Despite the very different densities of the two networks, Fig 6 shows that the matrices of link densities giving the mixing patterns of the different classes have a clear structural similarity, as was the case for the contact matrices emerging from the contact diaries. Although the values of each cell differ between the two matrices, the structure of classes and of blocks of classes of the contact patterns occurring between students in the high school is well mirrored in the network of declared friendships, with a very high similarity between the two matrices.

More in detail, 86% of the links in the symmetrized friendship network find a corresponding link in the network of contacts, while only 28% of the links of the contact network correspond to a friendship link. These numbers change if we restrict the contact data to stronger links, i.e., to contacts of larger aggregate duration: if we consider only links with an aggregate duration of more than 1 min (resp. 3 min) we find that 75% (resp. 62%) of the declared friendship links have a corresponding link in the contact network, while 45% (resp. 58%) of the contact network links correspond to friendships.

We investigate moreover in Fig 6(b) and Table 6 the diversity of the cumulative durations registered by the sensors for the reported friendships: we compare the cumulative distributions of the aggregate durations of contacts between pairs of students, distinguishing between students who both reported a friendship with each other, pairs of students with only one directed link of friendship reported, and pairs of students who did not report any friendship with each other. For reference, we show on the same graph the distribution of the aggregate durations of all contacts registered between the 134 students common to both networks. All distributions are broad: even pairs of students who have both reported a friendship link might have spent little time in contact. However, the aggregate duration of contacts of declared friends have a larger average and a broader distribution, especially if the friendship was reported by both (Wilcoxon tests reject the null hypothesis of equal distributions at 5% significance level). In particular, all links in the contact network with aggregate duration larger than a certain threshold (close to 2 hours and a half) correspond to a declared friendship. Thus, even if a reported friendship link can correspond to effective contacts of very different durations, the global network of friendships includes the most important contacts in terms of durations. We also note that both in- and out-degrees in the friendship survey network are positively correlated with the degree in the contact network: we obtain correlations of resp. 0.4 and 0.3, rising to 0.51 and 0.45 if only links with aggregate contact durations above a threshold of 1 min are taken into account in the contact network.

**Table 6:** Mean and standard deviation of distribution of aggregate durations for different sets of links (as in Fig 6(b)).

	All contact network links	Reciprocated friendship	Non-reciprocated friendship	No reported friendship
**Mean (s)**	926	3584	942	189
**Std. dev.**	3438	6714	3349	517
**Number of links**	1235	245	103	887

**Fig 7:**
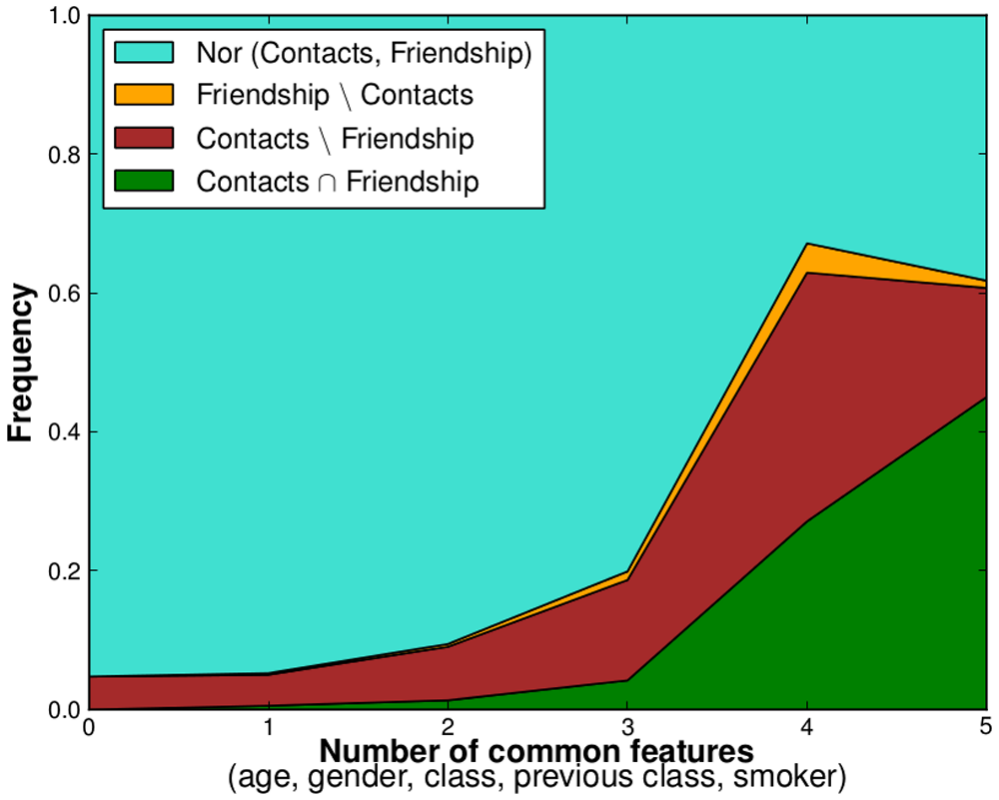
Fraction of friendship and contact links as a function of the number of features shared by two students.

We finally investigate in Fig 7 how the metadata can provide more intuition on the existence of both contacts and friendship links. Each pair of students can indeed share between 0 and 5 characteristics among age, gender, class, class in the previous year, and smoking behavior. If two students share less than 4 features, the largely most probable situation is that they did not have any contact and are not friends either. On the other hand, if they share 4 or 5 features, at least one link is found between them with higher probability. In particular, friendship relations are observed almost only between students sharing 4 or 5 features, especially if they did not have any contact. Contacts among non-declared friends on the other hand can also be found for pairs of students sharing few features, highlighting the more random character of such links.

#### Face-to-face contacts and Facebook links

We now perform a similar analysis as in the previous subsection, but focusing on a comparison between contact and Facebook data. As explained in the Methods section, only 17 students provided us with the network of their Facebook friends, so that we could not build the entire network of Facebook relationships between the students but rather work with a list of pairs of students (“known-pairs”) for which we know if they have a friendship relation on Facebook or not. The corresponding data set includes 4515 known-pairs involving 156 students, with 1437 Facebook links (and 3078 known pairs of students with no Facebook link). Moreover, these 156 students have 1118 links in the aggregated contact network. 52% of the Facebook links find a corresponding link in the contact network, and 67% of the 1118 links of the contact network are between Facebook friends.

The 17 students who gave access to their Facebook data were 9 in 2BIO3, 7 in MP and 1 in MP*2. The class repartition for the resulting 156 students was: 17 in 2BIO1, 14 in 2BIO2, 32 in 2BIO3, 26 in MP, 13 in MP*1, 20 in MP*2, 9 in PC, 13 in PC* and 12 in PSI*. As in the other data sets, Wilcoxon tests do not show any significant difference in the distributions of the contact properties of these students with respect to the others.

**Fig 8:**
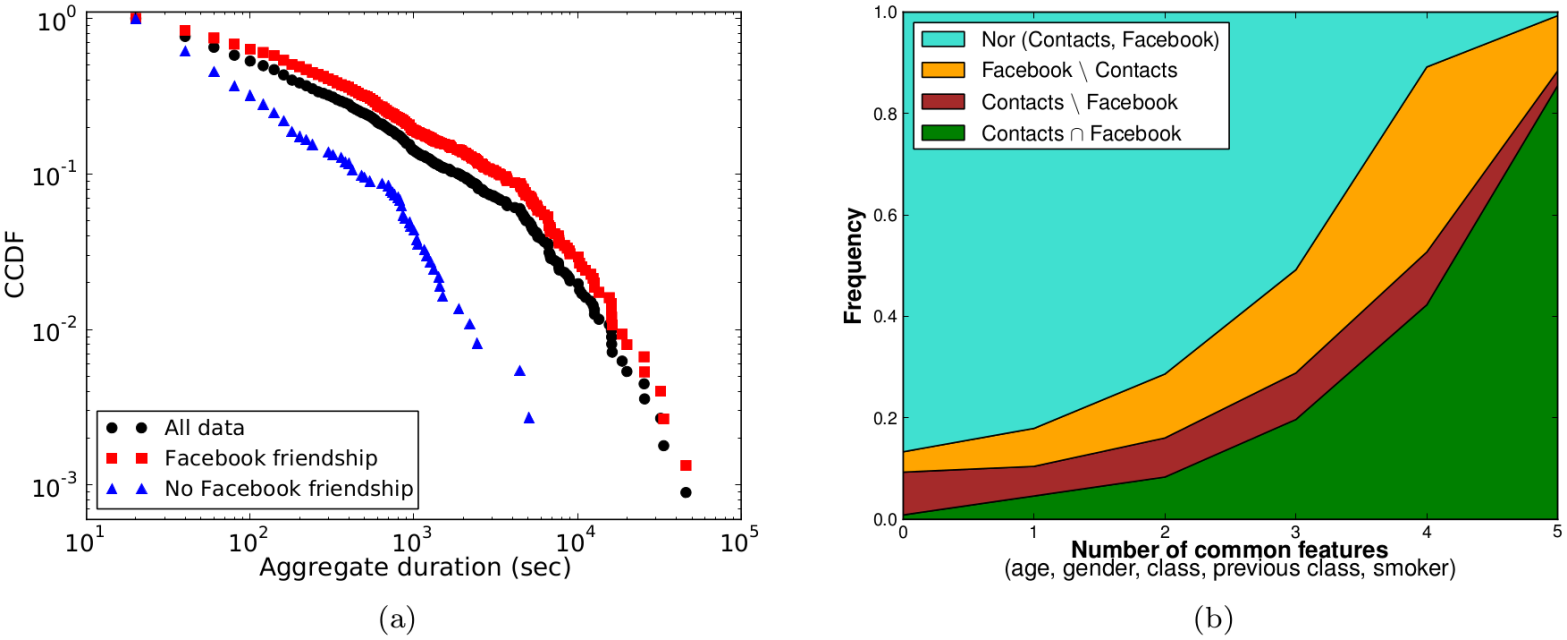
Contact vs. Facebook links. a) Distribution of aggregate durations for the different sets of links. b) Fractions of pairs of students belonging to specific groups (no link, link in both the contact network and Facebook, link in only one of the two) as a function of the number of common features.

More information about the aggregate durations of links present in the contact network and corresponding, or not, to a Facebook link is provided in Fig 8(a) and Table 7: both distributions are broad, as in the case of reported friendships (Fig 6), but the links between students who are not friends on Facebook have a clearly narrower duration distribution, and links with aggregate duration larger than a certain threshold correspond all to contacts between Facebook friends. The aggregate durations of contacts between students who are friends on Facebook display however a less broad distribution (which has also a smaller average) than the ones of students with a reciprocated reported friendship (compare with Fig 6(b)).

**Table 7:** Mean and standard deviation of the distributions of aggregate durations for different sets of links in the contact network.

	All data	Facebook friendship	No Facebook friendship
**Mean (s)**	917	1275	186
**Std. dev.**	3032	3634	464
**Number of links**	1118	750	368

This last point indicates a different nature of survey-reported and online friendships, which is further investigated by comparing Fig 8(b) with Fig 7: the number of shared features of a pair of students has a still strong but clearly smaller influence on the fraction of such pairs having a link in the contact network or on Facebook, and a substantial fraction of pairs of students with none or only one feature in common have a Facebook link. Facebook links which do not correspond to contacts are also observed between pairs of students with any number of shared features.

#### Contacts and friendship networks as a multiplex

Friendship relations, online friendship and face-to-face contacts can be combined to provide a more complete picture of the relationships between students in the high school. Each pair of students can be characterized by one, two, three or none of these three possible links, forming a multiplex network with three layers (strictly speaking, we have a multiplex set of nodes and links and not a network, as for the Facebook layer we do not have information about many pairs of nodes). We here perform a simple analysis of this multiplex, in which we consider only students who are part of all three corresponding data sets: they represent 82 nodes, 992 links in the contact network (aggregated over the whole study), 326 reported friendship links and 1026 Facebook links.

**Fig 9:**
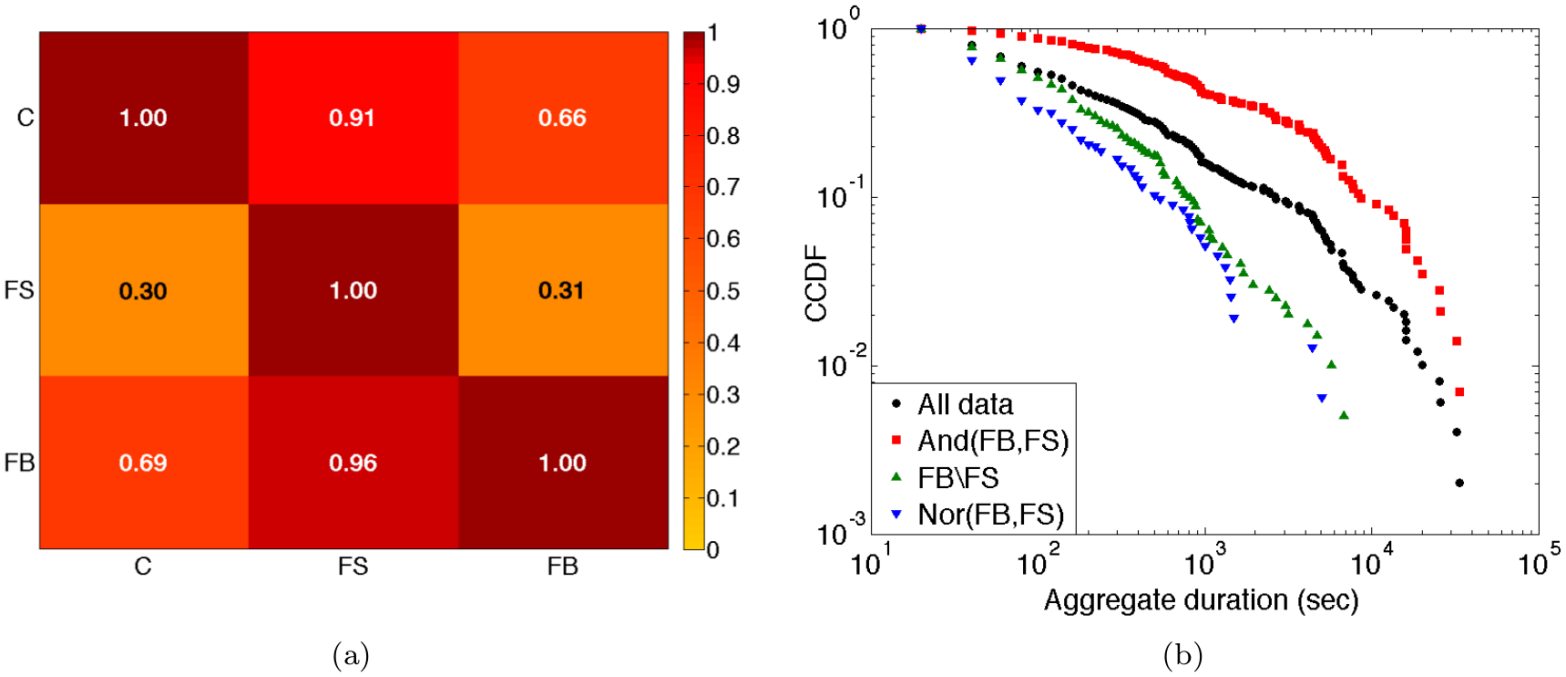
Multiplex Analysis. (a) Conditional probability to find a link in one layer (row index) given its existence in another one (column index); “C” stands for contact network, “FS” for friendship survey, “FB” for Facebook; (b) Distribution of aggregate durations in the contact network for different sets of links.

Fig 9(a) reports in a matrix form the conditional probability to observe a link between two students in a layer of the multiplex, given that the two students are linked in another layer [42]. The probabilities that a link is present in Facebook or that a contact was registered, given that a friendship has been reported, are very high. The probabilities that a friendship is reported if a contact has been registered or given that a Facebook link exists are much lower. Overall, contacts and Facebook links have similar properties in comparison with the reported friendships.

The strong difference between reported and Facebook links, already noted above, is emphasized by the results of Fig 9(b) and Table 8 showing the cumulative distribution of aggregate contact durations, as measured by the wearable sensors, for different sets of nodes pairs. Although all distributions are broad, important differences are present: the duration distribution is much broader for the pairs of students who are both reported friends and friends on Facebook; on the other hand, the distribution of the aggregate contact durations for pairs of students who are only friends on Facebook is much narrower than the distribution of all contact durations, and similar to the one obtained for the pairs of students who are neither friends on Facebook nor reported friends. Links of a duration above a certain threshold are observed only between reported friends. With respect to the length of registered contacts, having a link on Facebook is therefore not at all equivalent to being reported friends: if reported friendship is not also present, such a link tends to correspond to rather short face-to-face contacts. This emphasizes the need of an analysis taking into account the three layers and not only the online friendship one (note that many pairs of students have a link on Facebook but did not report a friendship relation, while the opposite is true only for 11 pairs of students).

**Table 8:** Mean and standard deviation of distribution of aggregate durations for different sets of links. Only 11 pairs of students have a reported friendship but no Facebook links, so that we do not give the corresponding statistics.

	All data	Facebook link and reported friendship	Facebook link only	No friendship
**Mean (s)**	1155	3304	342	184
**Std. dev.**	3549	6001	820	515
**Number of links**	992	285	395	301

## Discussion

In this article, we have presented an analysis of several data sets concerning interactions between students in a high school, collected both through a decentralized sensing platform based on wearable sensors and through diaries and surveys. The fact that these data sets were collected at the same time and in the same population allowed us to compare them and to quantify the overlap and complementarity of data sets of different nature. Collection of contact data and comparison of interactions of different nature are of interest for many purposes, including the information of data-driven models of relevance in epidemiology, as well as the investigation of human behaviour and social relations. The high school environment considered here represents one of the many contexts in which day-to-day contacts and interactions take place, and our work represents a step in the larger goal of gathering data sets describing human populations of different nature and in various contexts. Moreover, schools and high schools are of particular interest in the epidemiology of infectious diseases as contexts in which many contacts take place, leading therefore potentially to important spreading events, as well as in social sciences for investigations of how individuals evolve in young ages. Data can also be collected more easily thanks to the direct involvement of students in small scale scientific projects based on the collected data.

Data collected thanks to the wearable sensors developed by the SocioPatterns collaboration yielded a temporally resolved network of contacts between the 327 participating students. This network had features similar to data collected in the same context in previous years [24] and in other contexts [38, 39], with heterogeneous contact durations and inter-contact times (burstiness, a well known feature of human dynamics observed in a variety of systems driven by human actions [43]). Activity timelines showed repeated daily activity patterns, with most contacts occurring during class and lunch breaks, a feature of interest in the design of containment measures of infectious diseases [44]. The aggregated contact network structure was very far from a homogeneous mixing hypothesis and shaped by the division of students in classes, with a substructure of three groups of three classes. This substructure corresponded to a grouping of classes according to the studied field, but could also have been reinforced by the fact that the classrooms of each group were physically close in the high school. Overall, these results confirm the robustness of the properties of contact patterns measured in different years in a given context as found in [24].

Although the use of sensors to measure contact patterns has become more widely available and affordable in the last years, such deployments are not always feasible. Other methods, in particular based on contact diaries, have been and are still widely used. We have thus, in the same spirit as [11], compared the network of contacts reported in diaries by the students with the contact network measured by the sensors. We mostly confirmed the results of [11] and obtained some further insights:

- many students who accepted to participate to the data collection by wearing sensors did not fill the diary, probably due to the extra burden at the end of a school’s day; although this uneven spread of respondents and non-respondents had some impact on the resulting structure of the networks built from contact diaries, with some strongly underrepresented classes, no significant difference was observed between respondents and non-respondents with respect to their contact characteristics;
- similarly to the data of [10], not all contacts between two individuals were reported by both; when both students reported the contact, the approximate reported duration was most often the same;
- as found in [11], most short contacts detected by sensors were not reported in diaries, while the reporting probability was high for contacts with long enough aggregate durations; overall, the reporting probabilities were in-between the values reported in [10] and [11], respectively;
- the distribution of aggregate durations measured by the sensors were broad for contacts both reported and not reported in the diaries; the distribution was however broader for contacts reported, and all contacts of a long enough duration (as measured by sensors) were reported in the diaries;
- the contact durations reported by students tended to overestimate the durations measured by sensors; this outcome is in agreement with results from social studies about self-reported diaries biases stating that individuals tend to perceive the time spent in some activities (talk, work, play) differently from the reality [31] and frequently to overestimate it [45];
- despite the lower sampling in the diary data, the overall structure of the sensor contact network was recovered in the diary data, with a similar contact matrix. The density of the diary-based contact network was however much smaller due to the many short contacts not reported; as a result, paths between individuals appear longer in the diary-based contact network;
- we observed a correlation between the degrees of a node (number of distinct persons encountered) in both networks; when considering the diary-based contact network as directed, we observe that the degree measured by sensors is not correlated with the out-degree of a node (i.e., the number of contacts reported by the individual) but is correlated with its in-degree (i.e., the numbers of other persons reporting a contact with him/her). Interestingly, this indicates that, in terms of actual contacts, the overall picture of each individual *as reported by all the others* is more accurate than the records of the individual him/herself. Moreover, by dividing individuals into two groups according to the variability of their contact durations, we have shown that this effect is more pronounced for individuals with a particularly large heterogeneity of contact durations: an arbitrarily large number of their short contacts is then not recorded in their diaries, so that their degree in the sensor-based contact cannot be guessed from their own diaries, nor whether they are hubs or not.

Our results are in line with existing literature about the limits of self-reported surveys, which can stem from response style and response set biases [46]: The former include general distortions which do not depend on the specific content of the survey, such as *acquiescence* (the tendency to endorse all statements) and *extreme* or *central responding* (tendency to indicate the extreme or the central values in scale-rating surveys); the latter include biases strongly related to the survey topic such as the so-called *social desirability* (tendency to answer according to a community “desired” response). These biases can in particular lead to an overestimation of the durations and numbers of contacts [29]. Overestimation of the contact durations could also be related to the small number of people met (node degree), as suggested by [45] who found underestimation only for very large degrees (here the distribution of degrees of the contact networks have mean ∼ 9 and standard deviation ∼ 4.3).

Overall, diaries reporting contacts give therefore a different picture of the contact network measured by sensors in two respects: on the one hand, population sampling issues are much more severe; on the other hand, most short contacts are lost. However, a kind of ‘backbone’ of the contact network is reported, in which one can deduce the overall structure of the network and the most important contacts (in terms of duration) are present. As the present data and the ones of [11] concern similar contexts (high schools), it would be of great interest to confirm the robustness of these findings in other contexts. In cases such that collecting diary-based is easier than using wearable sensors, such knowledge on the differences between these two kinds of data could help obtain a more complete picture of the actual contact patterns.

Friendship relations represent another kind of data for which surveys are commonly used; the resulting data sets are a priori less prone to biases due to imperfect memory, and might also be easier to collect in a given population than contact diaries. Surveys might indeed be given and collected on a less constrained time frame than contact diaries, as friendships evolve on longer time scales than contacts. Similarly, online social networks might in some cases be easier to collect automatically. How much the networks of reported friendships or of online links correspond to or complement actual face-to-face contact networks is however not well known [25, 35]. We have therefore asked the participating students to fill in surveys to report their friendships in the high school, and to give us access to the networks Facebook links of their Facebook friends. As in the case of contact diaries, the data suffered from low sampling rates, showing the lesser acceptability of these requests (most probably because of the added burden for students with a large number of courses to follow) with respect to the use of wearable sensors. The uneven spread of respondents in different classes lead to an uneven sampling of different classes, but the contact properties of respondents and non-respondents were not statistically different. The network of reported friendship was much less dense than the contact network, as could be expected: most declared friends had at least one face-to-face contact during the data collection, while many contacts also occurred between pairs of individuals who did not report a friendship link. Despite this difference in the networks, the contact matrices of link densities between classes, as measured by the reported friendships, had a structure similar to the one deduced from the actual contacts. We also found differences in the properties of the contact network links between declared friends and between pairs of non-friends: links with an aggregate contact duration of any length are found between pairs of friends, but the distributions of aggregate durations is much narrower for individuals who are not friends. In fact, all contact network links of large enough duration occur between friends who both reported each other in the survey. In terms of network structure, the network of reported friendships gives information of quality similar to a contact diary with respect to the sensor-measured contact network (but lacks any estimation of the contact durations).

Interestingly, Facebook links yield a different picture than reported friendship ones. First, sampling issues prevent us from building a complete network of Facebook relationships. The probability that a contact is observed between two individuals, knowing that they are linked on Facebook, is also much smaller than the same probability conditioned on a reported friendship. In addition, the distribution of aggregate contact durations between individuals who are linked on Facebook but did not report a friendship to each other is much narrower than the one obtained for individuals who reported a friendship: overall, Facebook links seem to have a more ‘casual’ character than friendship links, in agreement with the intuition that Facebook links are easier to establish than real friendships.

**Limitations**. Our study has several limitations that are worth mentioning. As in similar data gathering efforts, only the contacts taking place within the reach of the radio receivers of the SocioPatterns infrastructure, i.e., within the high school premises, were recorded. Contacts occurring outside the high school are therefore missing from the data set. Not all students participated to the data collection, and some forgot to wear their sensors on some days, leading to data losses. Contacts with the outside population were also not recorded. Such contacts however are few for the population studied here, at least during workdays. The response rate for surveys and diaries was rather small; moreover, the corresponding sampling was uneven in different classes and possibly correlated, with probably groups of friends filling in the surveys and others not participating. How such uneven sampling affects the properties of the friendship network and contact diary network should be investigated further. We however checked that the respondents and non-respondents did not differ in terms of contact statistics as registered by the sensors. Friendship networks might also evolve at time scales larger than the study duration, so that it might be important to repeat measures and comparisons of the various networks at different times during the year. Finally, our data concern a specific environment and some results might differ in different contexts and for populations of different ages, in particular regarding the structure of friendships and the comparison between friendships and contacts. Further investigations in different contexts would be crucial to assess the robustness and generality of our results.

**Conclusion**. The collection of data with different methods has shown that, at least in the context of a high school in which students have a rather heavy work load (given they have competitive exams at the end of the year), the use of wearable sensors to gather data on contacts between individuals was more easily accepted than the burden of filling in contact diaries or surveys, and yielded therefore a better population sampling. Moreover, most short contacts detected by sensors were not reported in diaries and did not correspond to friendship relations, leading to much more dilute interaction networks based on diaries or surveys than on measured contacts. As a consequence, the distance between individuals in the former networks are overestimated, which could have strong consequences when using such data in data-driven models of dynamical processes (e.g., epidemic spreading). However, as all contacts of long enough durations were reported in the diaries and surveys, and as the diary-based and survey-based networks correctly revealed the structural organization of the population in classes, one could argue that the reported links carry enough information to feed data-driven models [32]. Future work will address this issue by performing numerical simulations of spreading processes on the various networks and comparing the outcomes. Another interesting avenue could be to understand how to combine data obtained with different methods to obtain a more complete picture of the interactions of a population: on the one hand, diaries could be used if not all individuals of a population wore sensors, to compensate for the resulting sampling bias; on the other hand, they could yield insights on how a given population (for which contacts are measured with sensors) enters in contact with the external world. Indeed, even when a high-resolution contact network has been measured among a given population and is used in a detailed data-driven simulation of epidemic spread for the evaluation of containment measures [44, 47], the interactions of the population of interest with the outside community are modeled in a simplistic way: using data from diaries and taking into account underreporting could help refine the model and test the robustness of the results.

## Supporting Information

S1 Supporting Information Pdf file containing supplementary figures and tables. (PDF)

S1 Dataset Dynamical contact list of the contacts between the students during 5 days in Dec. 2013, as measured by the SocioPatterns infrastructure. The file contains a tab-separated list representing the active contacts during 20-second intervals of the data collection. Each line has the form “t i j Ci Cj”, where i and j are the anonymous IDs of the persons in contact, Ci and Cj are their classes, and the interval during which this contact was active is [t −20s, t]. If multiple contacts are active in a given interval, multiple lines start with the same value of t. Time is measured in seconds.
(CSV)

S2 Dataset Directed network of contacts between students as reported in contact diaries collected at the end of the fourth day of the data collection. Each line has the form “i j w”, meaning that student i reported contacts with student j of aggregate durations of (i) at most 5 min if w = 1, (ii) between 5 and 15 min if w = 2, (iii) between 15 min and 1 h if w = 3, (iv) more than 1 h if w = 4.
(CSV)

S3 Dataset Directed network of reported friendships. Each line has the form “i j”, meaning that student i reported a friendship with student j.
(CSV)

S4 Dataset List of pairs of students for which the presence or absence of a Facebook friendship is known. Each line has the form “i j w”, where w = 1 means that students i and j are linked on Facebook, while w = 0 means that they are not.
(CSV)
