# Supplementary material for: Contact Patterns in a High School: A Comparison between Data Collected Using Wearable Sensors, Contact Diaries and Friendship Surveys
Source: PLoS One. 2015 Sep 1;10(9):e0136497. doi: 10.1371/journal.pone.0136497 (PMC4556655; doi:10.1371/journal.pone.0136497)
Supplement: S1 Supporting Information — (PDF) [file pone.0136497.s001.pdf]

Contact patterns in a high school: a comparison between data collected using wearable sensors, contact diaries and friendship surveys: Supplementary Information

Rossana Mastrandrea[1], Julie Fournet[1], Alain Barrat[1,2,*]
[1] *Aix Marseille Université, Université de Toulon, CNRS, CPT, UMR 7332, 13288 Marseille, France*
[2] *Data Science Laboratory, ISI Foundation, Torino, Italy*
* *E-mail: alain.barrat@cpt.univ-mrs.fr*

1 Properties of the contact network measured by wearable sensors

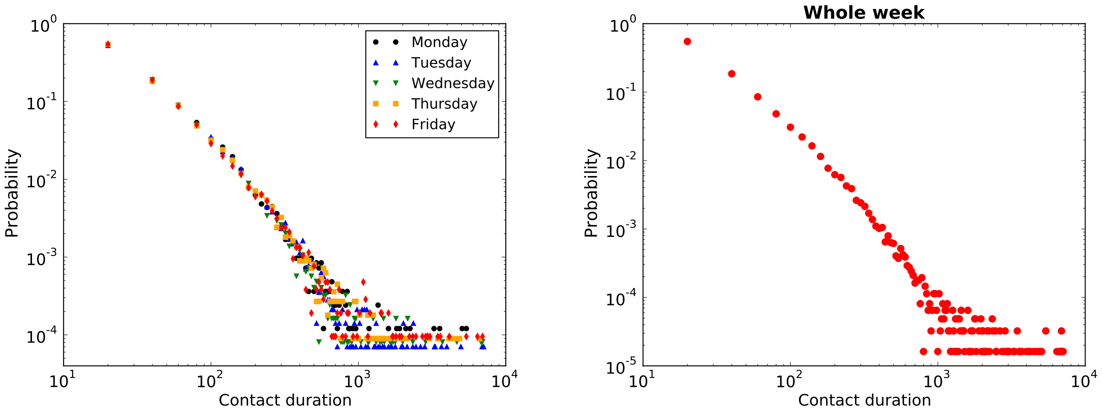

Fig. S1: **Distribution of contact durations in the temporal network of contacts measured by the SocioPatterns sensing platform.** The left panel shows the distributions of contact durations for each day of the week, the right panel for the whole data set.

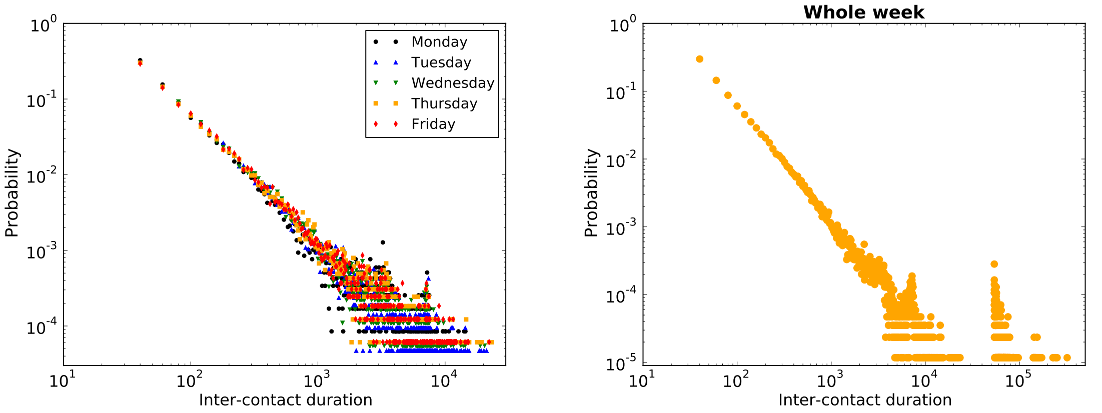

Fig. S2: **Distribution of inter-contact durations.** The left panel shows the distributions of time elapsed between successive contacts of a given pair of individuals in each day. The right panel displays the distribution measured on the whole data set. The peaks on the right correspond to inter-event durations including one or two nights.

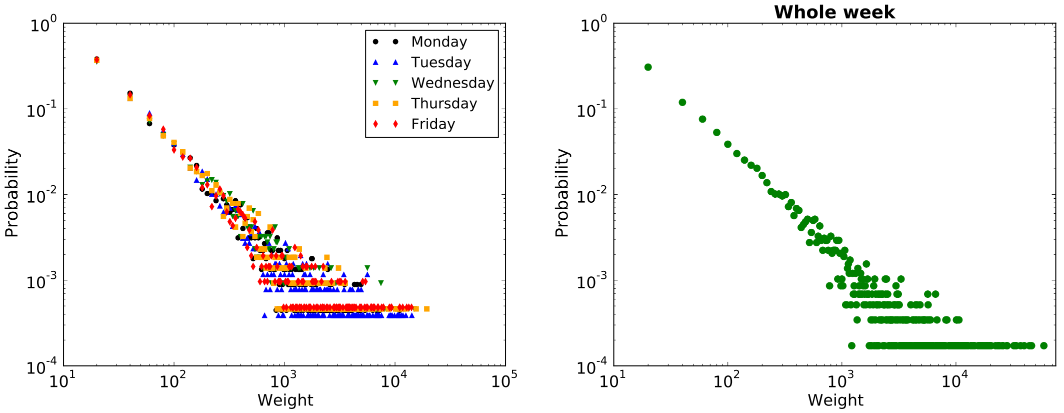

(a) (b)

Fig. S3: **Distribution of edge weights,** i.e., of aggregated contact durations between individuals, (a) in the daily aggregated contact networks, (b) in the contact network aggregated over the whole week.

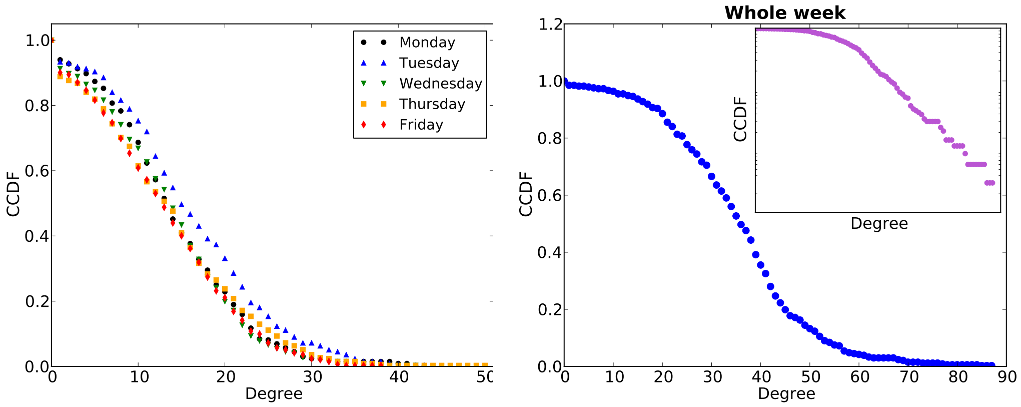

(a) (b)

Fig. S4: **Complementary cumulative distribution function of degrees,** i.e., of distinct individuals with whom a person has had contacts, (a) in the daily aggregated networks (b) in the contact network aggregated over the whole week. The inset shows the same distribution in lin-log scale.

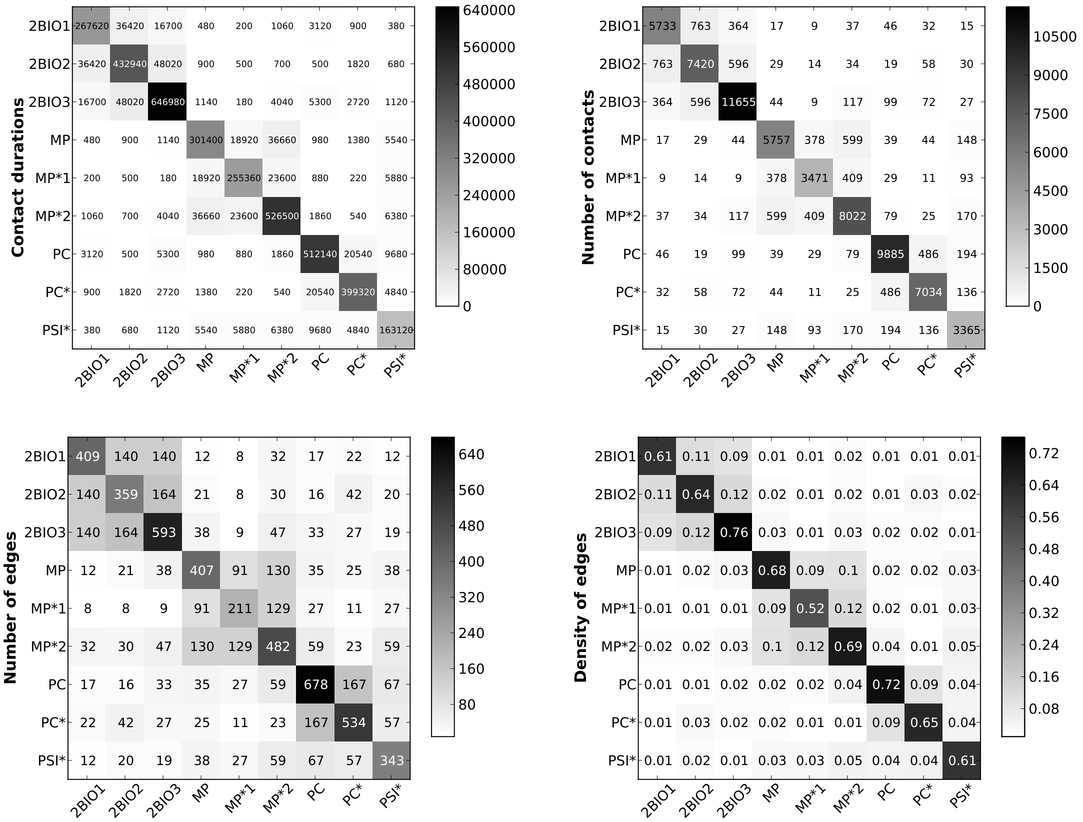

Fig. S5: **Contacts matrices** giving the cumulated durations in seconds and the numbers of contacts between classes (first row) and the numbers and densities of links between classes in the contact network (second row), for data aggregated over the whole week of data collection.

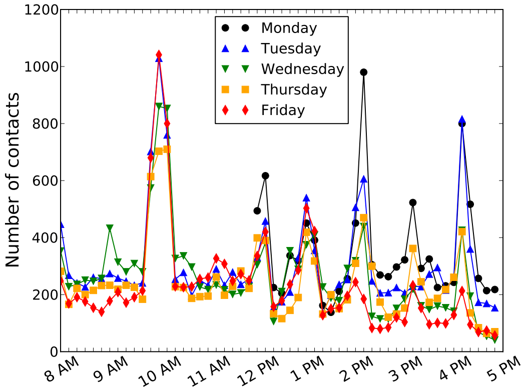

Fig. S6: **Activity timelines.** Number of contacts collected by the sensors per 10-minutes periods for each day.

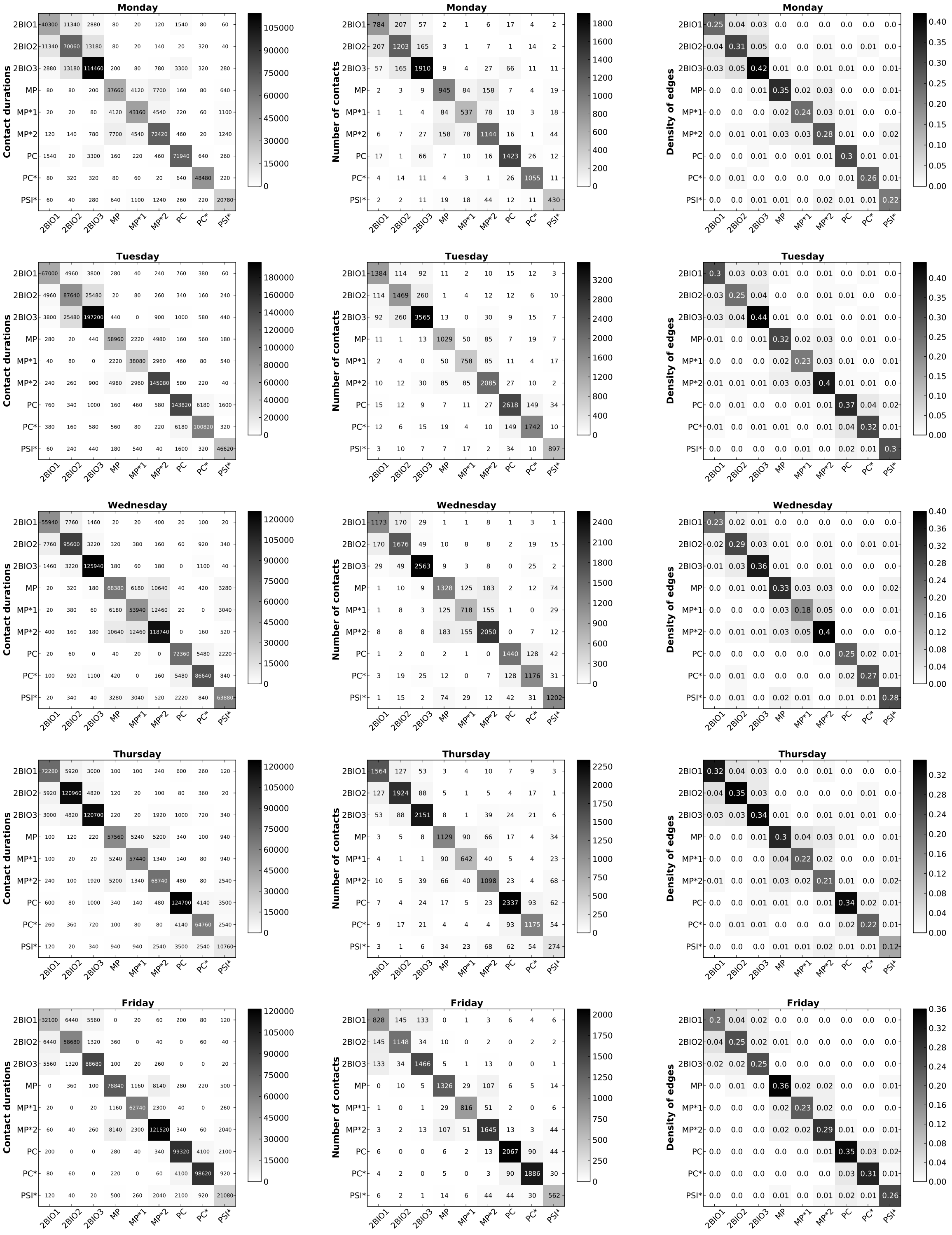

Fig. S7: **Contact matrices for each day of the week of study.** Contact matrices giving the cumulated durations of contacts (first column), the numbers of contacts (second column) and the densities of links (third column) between classes.

2 Comparison between the networks built from sensor data and from contact diaries

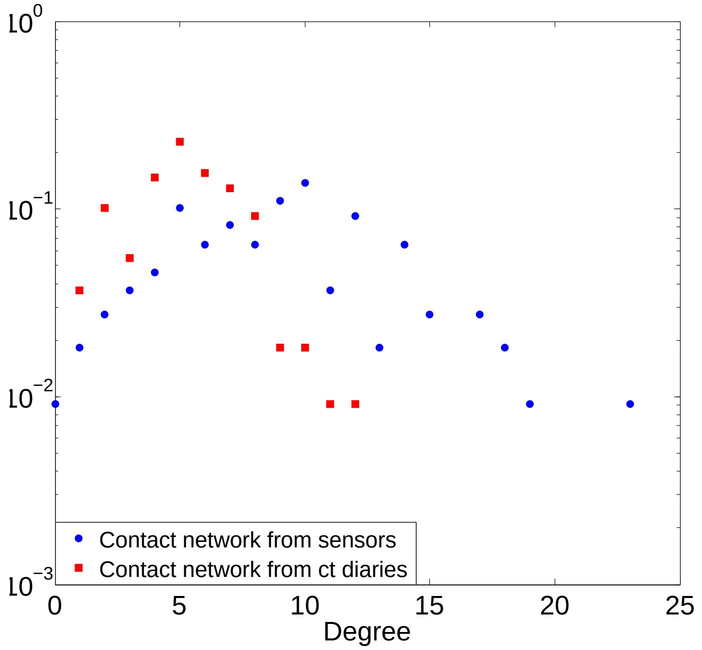

Fig. S8: **Degree distributions of the contact networks obtained by sensors and by contact diaries.**

Threshold	k_{in}	k_{out}
0s (488 links)	0.4* (0)	0.14 (0.16)
40s (328 links)	0.44* (0)	0.22* (0.02)
60s (263 links)	0.5* (0)	0.2* (0.02)
80s (221 links)	0.49*(0)	0.17 (0.08)
100s (203 links)	0.47* (0)	0.16 (0.08)

Tab. S1: **Sensors vs. contact diaries: node degree correlations.** Correlation between the degree of a node in the contact network built from the sensor data and the in- or out-degree of the same node in the network built from contact diaries. p-values are given in parenthesis. Each row corresponds to keeping only links with a minimum aggregate duration of contacts (Threshold) in the contact network built from sensor data.

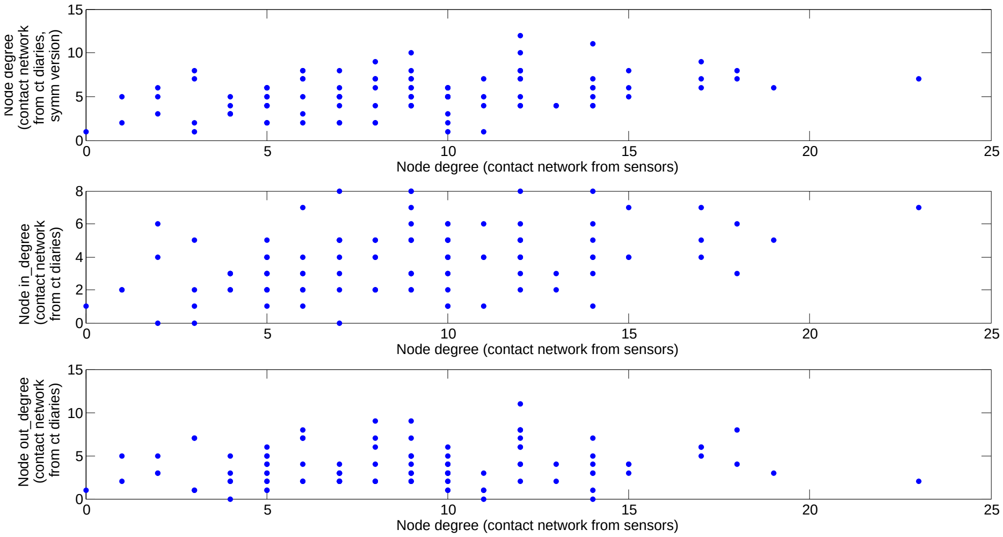

Fig. S9: **Comparison of the degree of individuals in the contact networks obtained by sensors and by contact diaries.** Scatterplot of the number of links of each node in both networks. As the network built from contact diaries is directed, we consider the degree in its symmetrized version (top), the in-degree (middle) and the out-degree (bottom), vs. the degree in the contact network obtained from sensor data.

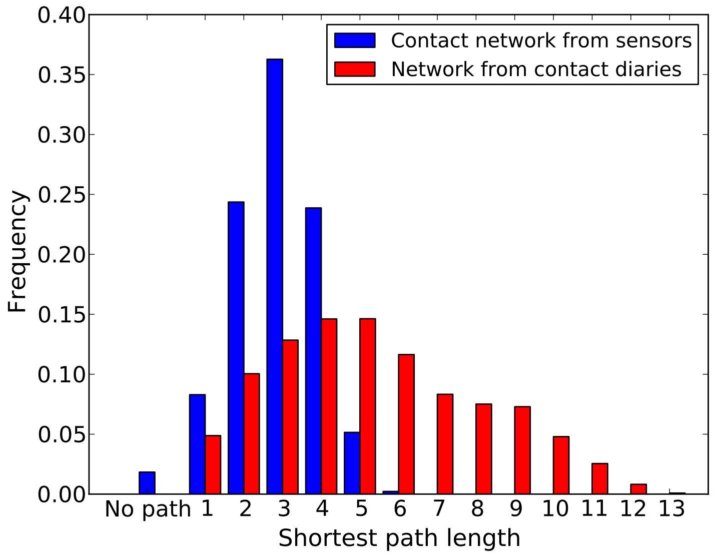

Fig. S10: **Sensors vs. contact diaries: shortest path length distribution** in the networks of contacts built using the sensor (blue) and the contact diary (red) data. "No path" corresponds to isolated nodes.

3 Comparison between sensor data and friendship-survey network

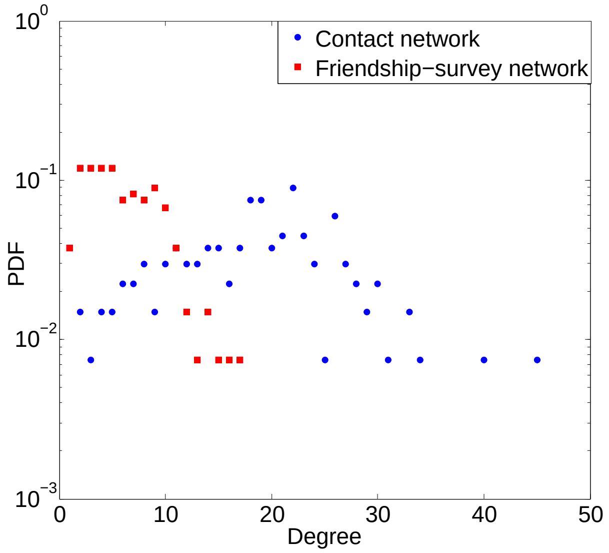

Fig. S11: **Sensors vs. friendship-surveys: degree distributions** in the contact network measured by the sensors and in the network built from the friendship surveys.

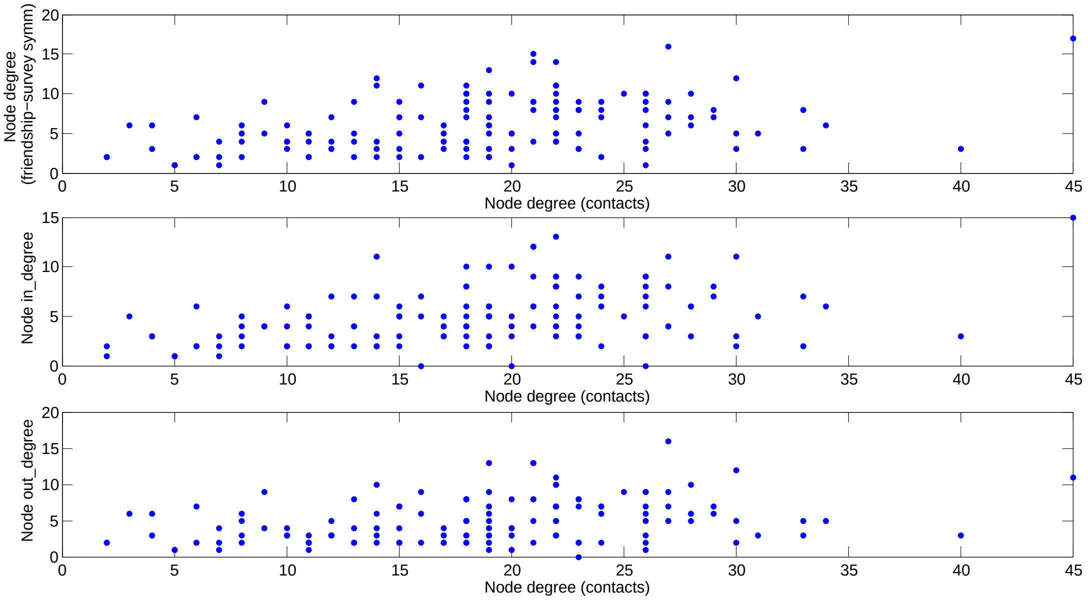

Fig. S12: **Comparison of the degree of individuals in the contact networks obtained by sensors and built from the friendship surveys.** Scatterplot of the number of links of each node in both networks. As the network of friendships is directed, we consider the degree in its symmetrized version (top), the in-degree (middle) and the out-degree (bottom), vs. the degree in the contact network obtained from sensor data.

**Threshold**	$\mathbf{k_{in}}$	$\mathbf{k_{out}}$
0s (1235 links)	0.42* (0)	0.34*(0)
40s (902 links)	0.51* (0)	0.44* (0)
60s (765 links)	0.51* (0)	0.45* (0)
80s (671 links)	0.53*(0)	0.47*(0)
100s (610 links)	0.52* (0)	0.47*(0)

Tab. S2: **Sensors vs. friendship-surveys: node degree correlations.** Correlations between the degree of a node in the contact network built from the sensor data and the in- or out-degree of the same node in the friendship network. p-values are given in parenthesis. Each row corresponds to keeping only links with a minimum aggregate duration of contacts (Threshold) in the contact network built from sensor data.
